# Transcriptome-Proteome Profiling in *Burkholderia thailandensis* during the Transition from Exponential to Stationary Phase

**DOI:** 10.1021/acs.jproteome.5c00223

**Published:** 2025-07-18

**Authors:** Ahmed Al-Tohamy, Fabrizio Donnarumma, Anne Grove

**Affiliations:** † Department of Biological Sciences, 5779 Louisiana State University, Baton Rouge, Louisiana 70803, United States; ‡ Department of Cell Biology, Biotechnology Research Institute, National Research Centre, Dokki, Cairo, 12622, Egypt; § Department of Chemistry, 5779 Louisiana State University, Baton Rouge, Louisiana 70803, United States

**Keywords:** *Burkholderia thailandensis*, mass spectrometry, metabolic adaptation, omics integration, protein−protein interaction network, proteomics, RNA-Seq, RpoS, transcriptomics

## Abstract

*Burkholderia thailandensis* is closely related to, and a surrogate for, highly pathogenic *Burkholderia* species. Like other bacterial cells, it commonly exists in the stationary phase, for instance, within a host cell. Understanding the molecular mechanisms that characterize the transition from exponential to stationary phases is therefore critical to understanding responses to stress or nutrient limitation. We present here an integrated transcriptomic and proteomic analysis of mRNA and protein abundance changes during entry into the stationary phase. We identified 928 differentially accumulating mRNAs and 832 differentially accumulating proteins. mRNAs encoding proteins involved in benzoate degradation and O-antigen nucleotide sugar biosynthesis were elevated in the stationary phase, whereas processes such as translation and flagellar biosynthesis were downregulated. Proteins related to fatty acid degradation and butanoate metabolism accumulated in the stationary phase along with proteins involved in the synthesis of secondary metabolites. Markedly downregulated proteins in the stationary phase included ribosomal proteins as well as the house-keeping iron–sulfur biogenesis proteins. An only modest correlation between transcriptome and proteome changes was seen, and the RpoS sigma factor was not significantly increased during the stationary phase; RpoS is typically abundant during the stationary phase and critical for expression of stress-response genes. Our data point to distinct adaptive mechanisms, possibly including post-translational regulation.

## Graphical abstract

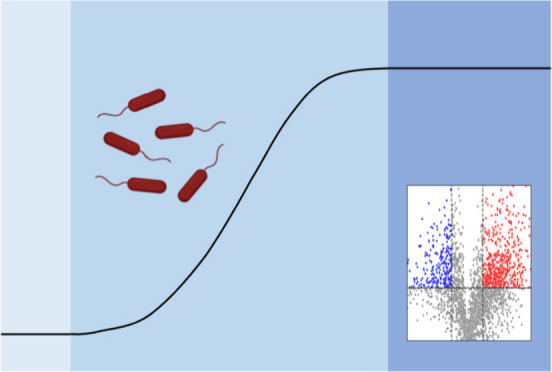

## Introduction

*Burkholderia* is a diverse genus of Gram-negative bacteria, and many species exhibit pathogenicity in plants and animals.^1^ Several of these microorganisms are well-known for their antibiotic resistance and ability to cause severe illness, particularly in immunocompromised individuals, such as cystic fibrosis patients.^2^ Other *Burkholderia* species hold biotechnological potential due to their capacity to degrade environmental pollutants.^3^ This dual nature has sparked growing interest in understanding their metabolic capabilities and antibiotic resistance mechanisms. *Burkholderia thailandensis*, although typically considered noninfectious to humans, serves as an excellent surrogate due to its close evolutionary relationship to pathogenic *Burkholderia* species, particularly *Burkholderia pseudomallei*, which causes melioidosis.^4,5^

Understanding the metabolic and regulatory pathway dynamics during different growth phases is crucial for understanding bacterial physiology. In nature, bacteria may experience nutrient-replete environments in which they can grow and divide (feast). However, they are even more likely to encounter nutrient-depleted or stressful conditions under which mere survival becomes paramount (famine). Such adverse conditions, which may, for instance, be encountered in a host environment, require the bacteria to adjust their gene expression, protein activity, and metabolic or developmental programs. In the laboratory, these conditions may be simulated by the transition from exponential growth to stationary phase.^6,7^ During an initial lag phase, which is characterized by metabolic activity without an increase in cell number, the bacteria adjust to their new environment, synthesizing essential proteins and metabolites required for subsequent growth. During the active growth phase, cells divide by binary fission, leading to a rapid increase in the population. This phase is marked by high metabolic activity; the population size doubles at regular intervals, resulting in exponential growth.^6^

During the stationary phase, the growth rate slows down due to nutrient depletion and the accumulation of waste products, with no net increase in population density.^6^ However, cells remain metabolically active and undergo significant physiological changes to adapt to a nutrient-limited environment. This phase is distinguished by extensive metabolic reprogramming, including the downregulation of protein synthesis, repression of aerobic metabolism, and the upregulation of alternative energy production pathways.^8^ Entry into the stationary phase is a carefully regulated process; one of the early events in Gram-negative bacteria is considered to be accumulation of the alternative sigma factor RpoS, which in turn governs the expression of genes encoding proteins required for survival under suboptimal conditions. Upregulation of RpoS has been reported to be a consequence of changes in transcription, translation, and protein stability.^9^

Amino acid starvation and other nutrient limitations also trigger the stringent response, which is characterized by accumulation of the hyperphosphorylated guanine nucleotides (p)­ppGpp. Their synthesis is, for example, triggered by the presence of uncharged tRNAs in the ribosomal A site.^10^ (p)­ppGpp then binds the RNA polymerase to affect promoter selectivity and to favor association of RpoS in preference to the housekeeping sigma factor, σ.^70^ In addition, (p)­ppGpp accumulation leads to elevated levels of RpoS.^11^ As a result, rRNA synthesis, ribosomal protein production, and DNA replication are downregulated, while amino acid biosynthesis is upregulated.^12^ Increased utilization of long-chain fatty acids as a carbon source is also characteristic of a stationary phase, and this utilization is promoted indirectly by RpoS.^11,13^

Some bacteria also increase production of secondary metabolites upon entry into the stationary phase. These compounds can serve various functions, including acting as antibiotics, siderophores, or signaling molecules.^14,15^ This shift toward secondary metabolism may reflect a competitive strategy as nutrients become scarce.

Regulation of gene expression features prominently as the bacteria adjust to changing environments.^16^ Bulk RNA sequencing (RNA-seq) has been extensively used to map such transcriptome changes. However, it is becoming increasingly apparent that post-transcriptional regulation is equally important during entry into the stationary phase. For example, in *Rhodobacter sphaeroides*, proteome changes often outpace and sometimes diverge from transcriptome changes as the bacteria enter the stationary phase.^17^

Here, we present an integrated view of transcriptome and proteome changes during the transition from exponential to stationary phases of *B. thailandensis*. These insights provide a foundational understanding of the adaptive mechanisms employed by *B. thailandensis* in response to stress or nutrient limitation.

## Experimental Procedures

### Bacterial Strains and Media

The *Burkholderia thailandensis* E264 strain, obtained from the American Type Culture Collection (ATCC), was cultivated in 2 × YT medium (16 g/L tryptone, 10 g/L yeast extract, 5 g/L NaCl; adjusted to pH 7.0). Cultures were incubated at 37 °C with continuous shaking at 250 rpm. An overnight culture was diluted 1:100 in 100 mL of fresh 2 × YT medium. The absorbance at 600 nm was recorded every 2 h for approximately 24 h in three biological replicates, with each absorbance representing an average of three technical replicates. Samples representing exponential phase cells were collected at an OD_600_ of 0.6 ± 0.05. The stationary phase cells were collected at an OD_600_ of 2.6 ± 0.05.

### RNA Sequencing (RNA-Seq)

#### Sample Preparation and RNA Extraction

For RNA sequencing, three biological replicates from both the exponential and stationary phases were used. A 2 mL portion of a 100 mL culture was collected, followed by centrifugation at 16,000 × g for 2 min. The cell pellets were washed twice with diethyl pyrocarbonate (DEPC)-treated water and then immediately frozen at −80 °C. RNA extraction was conducted using the Monarch Total RNA Miniprep Kit (New England BioLabs), following the manufacturer’s guidelines. The integrity of the extracted RNA was assessed by agarose gel electrophoresis. RNA was quantified using a NanoDrop 2000c spectrophotometer. An Agilent 2100 Bioanalyzer was used to obtain the RNA Integrity Number (RIN), selecting only samples with a RIN of 8 or higher for further processing.

#### Library Preparation and High-Throughput Sequencing

Library preparation and high-throughput sequencing was performed by Novogene (https://www.novogene.com). Library preparation was done using the Illumina TruSeq Stranded mRNA Library Prep Kit, adhering to the manufacturer’s instructions. Ribosomal RNAs were depleted from the total RNA, followed by ethanol precipitation. The RNA was then fragmented, and the first strand of cDNA was synthesized using random hexamer primers. In the synthesis of the second strand of cDNA, dUTPs were replaced with dTTPs in the reaction buffer. The library was further processed through end repair, A-tailing, adapter ligation, and size selection. Uracil-Specific Excision Reagent (USER) enzyme digestion was applied, followed by amplification and purification. After preparation, the libraries were quantified using Qubit and real-time PCR, and a bioanalyzer was used for the determination of size distribution. Libraries were pooled, high-throughput sequencing was executed by utilizing a paired-end read configuration on the Illumina NextSeq 500 platform, and Fastq files were generated.

#### Differential Gene Expression Bioinformatics Data Analysis

The bioinformatics pipeline started with a quality assessment using FastQC.^18^ The Trim Galore toolkit^19^ was then applied to remove sequencing adapters and unidentified bases (N) and to trim bases of low quality (phred score cutoff = 20). MultiQC was used to assess the quality of the processed data set.^20^ For alignment purposes, the reference genome of *B. thailandensis* was retrieved from the *Burkholderia* Genome Database (https://www.burkholderia.com).^21^ The genome was indexed by using SAMtools. Subsequent alignment of the high-quality reads to this reference genome was performed using the HISAT2 aligner.^22^ Postalignment, SAMtools was utilized to construct SAM and BAM files from the aligned reads that were used for further analysis. Gene expression quantification was conducted using FeatureCounts.^23^ The differentially expressed genes between exponential and stationary phases were identified using DESeq2 ^24^ based on the count matrix obtained. Genes were considered significantly differentially expressed if they exhibited a log2-fold change of ≥ 2 or ≤ −2 and if they met the statistical significance threshold with a false discovery rate (FDR) of <0.05. This FDR value was adjusted for multiple testing using the Benjamini-Hochberg procedure.^25^

#### Data Visualization

Heatmaps were constructed using the ComplexHeatmap package in R.^26^ Log2-fold change values were converted to z-scores, normalizing gene expression across samples. Hierarchical clustering was applied to group genes with similar expression patterns. To identify and visualize differentially expressed genes, Volcano plots were generated using the Plotly Express library in Python.^27^

### Quantitative Mass Spectrometry Proteomics

#### Protein Extraction, Tandem Mass Tag (TMT) Labeling, and Fractionation

We followed the filter-aided sample preparation (FASP) method^28^ with the following modifications. Forty milliliters of the 100 mL bacterial culture were centrifuged at 17,800 × g for 5 min, followed by two washes with phosphate buffered saline (PBS). The bacterial pellets were stored at −80 °C. Cell pellets were thawed on ice, and protein extraction was carried out by the addition of 1 mL of a lysis buffer (2% SDS in 100 mM tetraethylammonium bicarbonate (TEAB), pH 8.5). The cell pellets were sonicated twice using a Branson SFX250 Sonifier at 35% output at 10 s pulse with 1 min gap in between. Homogenized samples were centrifuged at 8,600*g* for 5 min at 4 °C. Protein concentration of the supernatant was determined using the Pierce bicinchoninic acid (BCA) Protein Assay Kit (Thermo Fisher Scientific).

Proteins (100 μg in total) were first reduced with 50 mM DTT and then alkylated with iodoacetamide. Subsequent overnight digestion was performed with LysC/Trypsin (Thermo Fisher Scientific) at 37 °C. TMTsixplex labeling was conducted following the manufacturer’s protocol (Thermo Fisher Scientific). Following labeling, the six TMT-labeled peptide samples (three representing the exponential phase and three from the stationary phase) were pooled and subsequently fractionated into six fractions using strong cation exchange stage tips (AttractSPE Disk, Affinisep, Le Houlme, Normandy, France). The 6 fractions were dried and resuspended in 0.1% formic acid. Detailed procedures are provided in the Supporting Information.

#### Data Acquisition

Mass spectrometry analysis was conducted on a Q-Exactive orbitrap mass spectrometer (Thermo Scientific, Waltham, MA) coupled to an Ultimate 3000 RSLC liquid chromatography system (Thermo Scientific). A binary mobile phase system was employed both for sample loading and trapping as well as for analytical separation. Sample injection was set to 5 μL, and each fraction was resuspended in 10 μL of 0.1% formic acid. The mobile phase composition was as follows: A = LC-MS grade H_2_O with 0.1% formic acid; B = 100% acetonitrile with 0.1% formic acid. The sample was loaded by connecting the capillary carrying the tryptic peptides to an Acclaim PepMap 100 C18 trap cartridge (0.3 × 5 mm, 5 μm particle size, 100 Å pore size) and transferred using a mobile phase composition of 2% B over the course of 5 min at a flow rate of 20 μL min^–1^. Separation was carried out on an Acclaim PepMap 100 C18 nanoLC column (0.075 × 250 mm, 3 μm particle size, 100 Å pore size) using a linear gradient from 6% to 35% mobile phase B over the course of 120 min at a flow rate of 300 nL min^–1^. The mass spectrometer was operated in data-dependent acquisition with a loop count of 10. Full MS parameters were as follows: resolution = 70,000, automatic gain control (AGC) = 1 × 10^6^, scan range = 300–1600 *m*/*z*, and maximum injection time = 30 ms. The data-dependent acquisition parameters were as follows: resolution = 35,000, AGC target = 5 × 10^4^, isolation window = 2 *m*/*z*, maximum injection time = 50 ms, and normalized collision energy = 30. Additional settings included: dynamic exclusion = 60 s, peptide match = preferred, excluded isotopes = on, charge exclusion = unassigned +7, +8, and > +8 charges, and maximum AGC target = 500, which resulted in an intensity threshold of 10,000.

#### Proteomics Data Analysis

The proteomics experiments yielded raw mass spectra, which were subjected to data processing using Proteome Discoverer software.^29^ This encompassed peak picking, spectral alignment, and peptide identification. For peptide identification, SEQUEST HT and MASCOT search engines were used. These engines matched the acquired spectra against the *Burkholderia thailandensis* protein database, which was obtained from UniProt (5,562 entries are specified in the UniProt database for *B. thailandensis* E264).^30^ To avoid false positive protein identification, only proteins for which two peptides were identified, of which one unique, were included. Quantification of protein abundances relied on the measurement of TMT reporter ion intensities. Proteins were considered significantly differentially expressed if they exhibited a log2-fold change of ≥0.5 or ≤ −0.5, and if they met the statistical significance threshold with a P-value of <0.05.

#### Data Visualization

Principal Component Analysis (PCA) was conducted using the PCA function from the sklearn.decomposition module in Python.^31^ The analysis was performed on standardized proteomic data to reduce the dimensionality and highlight the variance between the experimental conditions. The proteomic data were standardized using z-score normalization, where each protein abundance value was transformed by subtracting the mean and dividing the mean by the standard deviation across all samples. This ensures that all features contribute equally to Principal Component Analysis (PCA), preventing bias from proteins with inherently larger expression ranges. The PCA results were visualized using matplotlib.^32^ The Volcano plot was generated as described above.

### Protein–Protein Interaction (PPI) Network Construction

To construct the protein–protein interaction (PPI) network for *B. thailandensis*, the relevant protein FASTA files were retrieved from the UniProt database.^30^ Since *B. thailandensis* is not available in the STRING available organisms,^33^ we generated a custom reference proteomic data set using the STRING server based on this FASTA data. The differentially upregulated proteins were then used to construct the PPI network.

PPI data were sourced from the STRING database. The data set included interactions with combined confidence scores, reflecting the likelihood of functional associations between protein pairs. Further analysis of the PPI network was performed using custom Python script using NetworkX.^34^ We calculated each protein number of interactions, average interaction score, degree centrality, betweenness centrality, closeness centrality, eigenvector centrality, clustering coefficient, interacting protein list, cluster numbers, and average shortest path length. In the PPI, nodes represent proteins and edges represent interactions between proteins. To identify functional modules within the PPI network, the Louvain method for community detection was employed.^35^

### KEGG Pathway Changes and Gene Ontology (GO) Analysis

Gene Set Enrichment Analysis (GSEA) was performed to identify significantly regulated pathways using the KEGG database.^36^ Pathways were identified using the fgsea package in R.^37^ Pathway enrichment results were visualized using custom scripts to generate bubble plots, displaying the normalized enrichment score (NES), −log10­(p-value), and counts of genes/proteins.

Selected pathways were further visualized using the Pathview package in R.^37^ DEGs/DEPs, represented as log2-fold change (log2FC) values, were mapped onto KEGG pathways. Custom color schemes were applied, with upregulated genes shown in red and downregulated genes in blue, where the intensity of the color correlates with the magnitude of the expression changes.

GO Enrichment Analysis was conducted to identify overrepresented GO terms within DEGs and DEPs. GO terms were categorized into Biological Process (BP), Molecular Function (MF), and Cellular Component (CC) categories. The analysis was performed using custom Python and R scripts to assess the enrichment of GO terms. The enrichment results were filtered to include only those terms with a p-value <0.05. The enriched GO terms for each category were visualized using bar plots generated with ggplot2,^38^ where the *x*-axis represents the count of genes, and the *y*-axis represents the GO terms with the color scheme reflecting the level of statistical significance. Specifically, more intense red colors represent higher p-values, indicating lower statistical significance, while more intense blue colors represent lower p-values, indicating higher significance.

### Integration of RNA-seq and Proteomics Data

To integrate the RNA-Seq and proteomics data, gene-protein mapping was performed by using curated annotations from the UniProt database. The DEGs and DEPs were merged based on their respective gene or protein identifiers using a custom Python script. The integrated data set was then analyzed to identify genes and proteins with opposite or consistent regulation between RNA-Seq and proteomics data.

Pearson correlation^39^ analysis was performed to assess the relationship between RNA-Seq and proteomics data. Log2-fold changes from both data sets were normalized using the StandardScaler function from the sklearn library.^31^ Correlation coefficients and p-values were calculated using the scipy library.^40^ Scatter plots with regression lines were generated to visualize correlations, and density plots were created to compare the distributions of normalized log2-fold changes. Violin plots were used to compare the distribution of normalized log2-fold changes between the RNA-Seq and proteomics data sets. For visualization, seaborn^41^ and matplotlib^32^ libraries were used. For detailed information on statistical methods, please see the Supporting Information.

### RpoS Sigma Factor Analysis

#### RT-qPCR

An overnight culture of *B. thailandensis* was diluted 1:100 and subcultured in 2 × YT medium, and samples were collected at various OD_600_ values (0.1, 0.2, 0.4, 0.6, 1.1, 1.4, 1.6, 1.9, 2.2, 2.4, 2.6, 2.8, and 3.0). A 2 mL portion of the cell culture was pelleted, washed twice with autoclaved DEPC-treated water, and stored at – 80 °C. RNA was extracted utilizing the Monarch total RNA miniprep kit (New England Biolabs, Ipswich, MA) according to the manufacturer’s protocol. RNA was electrophoresed on agarose gels to ascertain the integrity. Quantitative RT-PCR was performed using Luna one-step universal master mix (New England Biolabs). Data represent means (±SDs) from biological triplicates (each determined from technical triplicates) using the comparative threshold cycle (*C*_*T*_) method (2^–ΔΔ*CT*^) for which *hgprt* (*BTH_I1148*) was used as a reference gene. The *C*_*T*_ values for *hgprt* were constant under the conditions used for these experiments. The primer sequences can be found in Supplemental Table S9.

#### Comparison of Regulons

The reported RpoS regulons from *B. pseudomallei* and *E. coli* were compared. Both reported regulons were based on 2D gel electrophoresis, followed by protein identification by mass spectrometry. For *B. pseudomallei*, 58 unique differentially expressed proteins were identified.^42^ The RpoS regulon of *E. coli* included 35 proteins.^43^ BlastP searches were used to identify homologous proteins in *B. thailandensis*.^44^

## Results

### Transcriptome Profiling

The growth of *B. thailandensis* was monitored, and cells representing exponential growth (OD_600_∼0.6) and stationary phase (OD_600_∼2.6) were collected for transcriptome profiling (Figure 1A). For each condition, cells were collected from three independent cultures (three biological replicates). Differentially expressed genes (DEGs) exhibiting a significant log2-fold change greater than |2| were primarily examined to evaluate transcriptome changes occurring on entry into the stationary phase. Based on this cutoff, we identified 928 DEGs, of which 564 were upregulated and 364 were downregulated (Figure 1B). The top 20 up- or downregulated genes are shown in Table 1; the entire data set is included in Supplemental Table S1. Hierarchical cluster analysis of DEGs visualized using a heatmap reflects the clustering of samples with similar expression patterns (Figure 1C).

**Figure 1:**
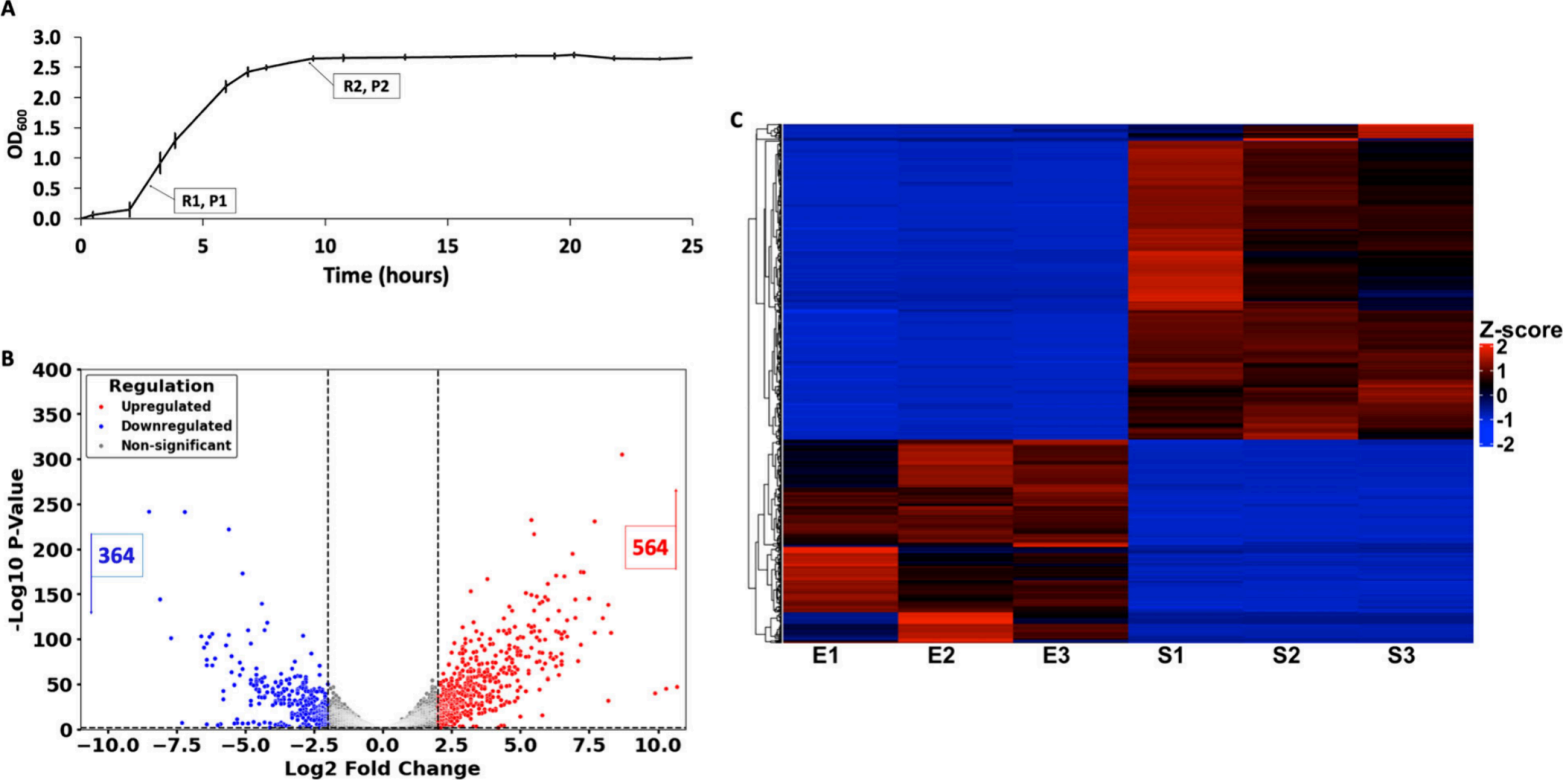
**Growth curve and gene expression analysis.** A. Growth curve of *B. thailandensis*: lag phase (OD_600_ ∼ 0.0–0.2), exponential growth (OD_600_ ∼ 0.2–2.4), and stationary phase (OD_600_ ∼ 2.5–2.7). Error bars represent the standard deviation from three biological replicates. Labels R1 and P1 indicate the point where cells were collected for RNA and protein isolation during the exponential phase, while R2 and P2 indicate the corresponding point for the stationary phase. B. Volcano plot of RNA-seq data. Significantly increased mRNAs in stationary phase (564) are colored red and reduced mRNAs (364) in blue. The horizontal dashed line hugging the *x*-axis represents a p-value of 0.05, while the vertical dashed lines indicate log2-fold changes of |2|. C. Heatmap of gene expression changes in exponential and stationary phase. Red indicates higher expression, and blue indicates lower expression. Hierarchical clustering on both gene and sample axes is included to display patterns of gene expression coregulation. Samples E1, E2, and E3 represent three biological replicates for the exponential phase, while samples S1, S2, and S3 represent three biological replicates for the stationary phase.

**Table 1:** Top Differentially Expressed Genes on Entry into the Stationary Phase

**Gene ID**	**Protein ID**	**Log2 Fold Change**	**Log2 Fold Change Standard Error**	**Regulation**	**Gene Name**
BTH_II0489	Q2T810	10.7	0.74	Up	Ortho-halobenzoate 1,2-dioxygenase beta-ISP protein OhbA
BTH_II0488	Q2T811	10.3	0.73	Up	Ortho-halobenzoate 1,2-dioxygenase alpha-ISP protein OhbB
BTH_II0490	Q2T809	9.9	0.74	Up	Rieske family iron–sulfur cluster-binding protein
BTH_II1638	Q2T4R7	8.7	0.23	Up	H-type lectin domain protein
BTH_II0483	Q2T816	8.3	0.38	Up	Muconolactone delta-isomerase
BTH_II0484	Q2T815	8.2	0.33	Up	Catechol 1,2-dioxygenase
BTH_II0491	Q2T808	8.2	0.70	Up	Ferredoxin reductase
BTH_II0698	Q2T7F2	8.1	0.21	Up	Hypothetical protein
BTH_II2182	Q2T379	8.0	0.34	Up	DUF1842 domain-containing protein
BTH_II2183	Q2T378	7.7	0.35	Up	DUF1842 domain-containing protein
BTH_II0485	Q2T814	7.7	0.24	Up	Muconate cycloisomerase
BTH_II2184	Q2T377	7.5	0.29	Up	DUF1843 domain-containing protein
BTH_II0722	Q2T7D0	7.3	0.26	Up	DUF1842 domain-containing protein
BTH_II0564	Q2T7T5	7.2	0.35	Up	BarB2 (barbamide)
BTH_I2307	Q2SW71	7.2	0.26	Up	DUF4189 domain-containing protein
BTH_II0563	Q2T7T6	7.1	0.38	Up	Peptide synthetase
BTH_II0718	Q2T7D4	7.1	0.18	Up	Hypothetical protein
BTH_II1925	Q2T3Y0	7.0	0.30	Up	Chitin binding domain-containing protein
BTH_I0617	Q2T0 × 5	7.0	0.27	Up	DUF4142 domain-containing protein
BTH_II0542	Q2T7 V7	6.9	0.23	Up	Mannose-1-phosphate guanylyltransferase (cepacian)
BTH_I1854	Q2SXG4	–9.1	0.22	Down	Nitrate reductase (quinone)
BTH_I1853	Q2SXG5	–8.9	0.17	Down	Nitrate reductase, beta subunit
BTH_I1855	Q2SXG3	–8.6	0.21	Down	Nitrate/nitrite transporter
BTH_I1852	Q2SXG6	–8.5	0.26	Down	Nitrate reductase, delta subunit
BTH_I1856	Q2SXG2	–8.1	0.32	Down	Nitrate/nitrite transporter
BTH_I3196	Q2STQ9	–7.7	0.36	Down	Flagellin
BTH_II0881	Q2T6 × 1	–7.3	1.39	Down	Copper-containing nitrite reductase
BTH_I1851	Q2SXG7	–7.2	0.22	Down	Respiratory nitrate reductase, gamma subunit
BTH_I0243	Q2T1Z8	–6.6	0.30	Down	Flagellar hook protein FlgE
BTH_I3168	Q2STT7	–6.5	0.32	Down	Flagellar biosynthesis protein FlhF
BTH_I0246	Q2T1Z5	–6.4	0.36	Down	Flagellar L-ring protein
BTH_I0244	Q2T1Z7	–6.4	0.35	Down	Flagellar basal-body rod protein FlgF
BTH_II0880	Q2T6 × 2	–6.4	1.42	Down	Sulfatase-modifying factor enzyme domain-containing protein
BTH_I0245	Q2T1Z6	–6.4	0.31	Down	Flagellar basal-body rod protein FlgF
BTH_I3197	Q2STQ8	–6.3	0.29	Down	Flagellar hook-associated protein 2 (HAP2)
BTH_I3167	Q2STT8	–6.2	0.35	Down	Flagellar biosynthesis protein FlhG
BTH_I0242	Q2T1Z9	–6.2	0.28	Down	Basal-body rod modification protein FlgD
BTH_I0197	Q2T244	–6.1	0.32	Down	Flagellum-specific ATP synthase
BTH_II0879	Q2T6 × 3	–6.0	1.48	Down	SCO1/SenC family protein
BTH_II0341	Q2T8F8	–5.9	1.27	Down	Ribosomal protein L15

To evaluate the biological functions of the DEGs, they were functionally categorized using the Kyoto Encyclopedia of Genes and Genomes (KEGG) pathway classification (Figure 2A and Supplemental Table S2). The top pathways involved in bacterial adaptation to the stationary phase were selected. Significantly downregulated pathways in the stationary phase included nitrogen metabolism, chemotaxis, and flagellar assembly, and genes encoding two-component systems were also generally downregulated. Upregulated pathways included benzoate degradation and the biosynthesis of nucleotide sugars.

**Figure 2:**
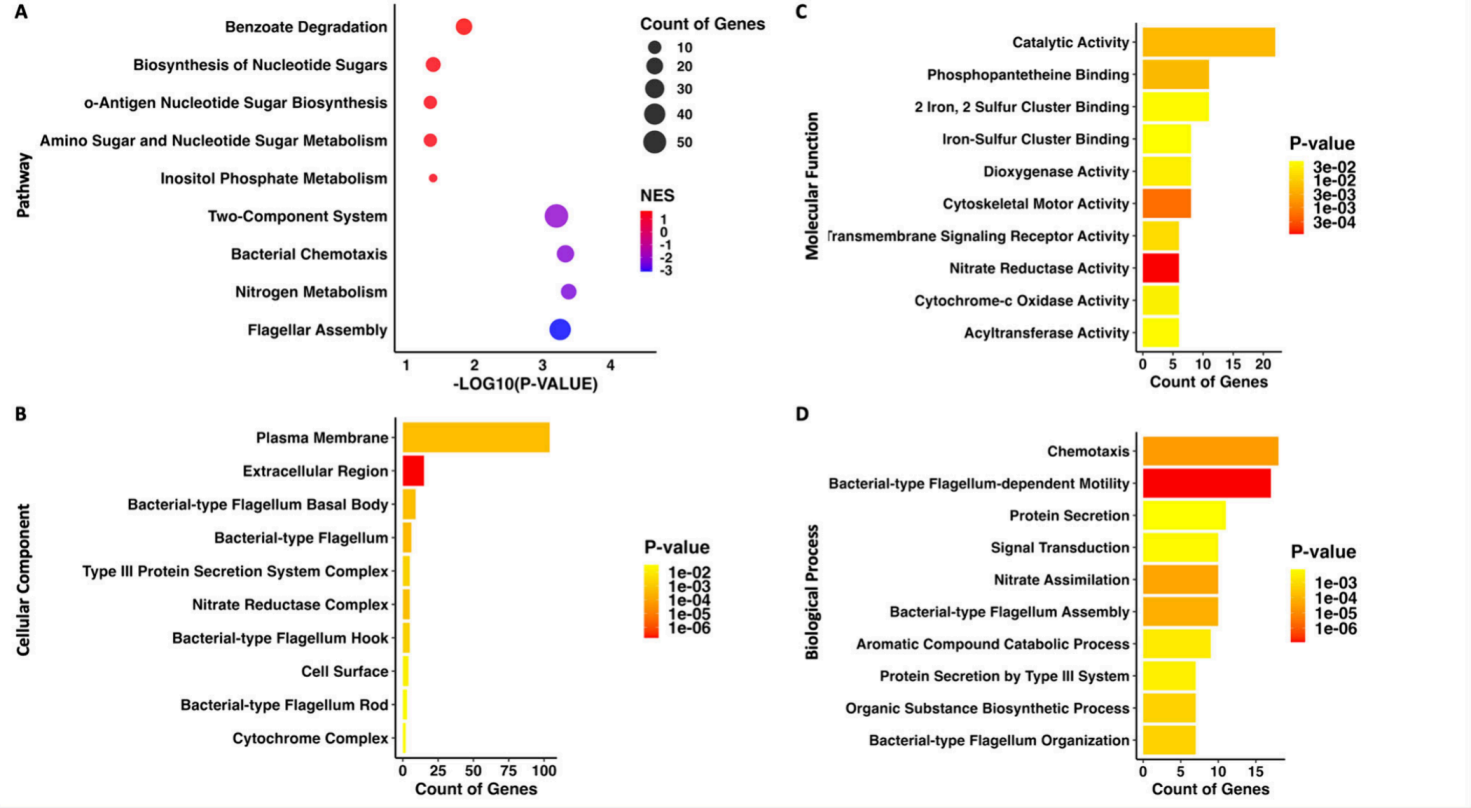
**RNA sequencing KEGG pathways and gene ontology.** A. Bubble plot illustrating significantly regulated KEGG pathways during the stationary phase compared to the exponential phase. Each pathway is represented by a circle, with the size of the circle indicating the count of DEGs in the pathway. The *x*-axis represents the −log10­(p-value) of the change. The color of the circles indicates the Normalized Enrichment Score (NES), with red shades representing upregulated pathways (NES closer to 1) and blue shades representing downregulated pathways (NES closer to −3). B.-D. Bar plots showing the enriched Gene Ontology (GO) terms among differentially expressed genes for Cellular Component, Molecular Function, and Biological Process, respectively. The *x*-axes show the observed gene count associated with each GO term. The color of each bar corresponds to the calculated p-value, with more red colors representing higher levels of enrichment significance (lower p-value).

Gene Ontology (GO) annotations were identified to provide a framework for describing altered functions. In the Cellular Component category, by far the largest category was plasma membrane, while categories also represented in the KEGG pathway analysis were also represented, including bacterial-type flagellum and the nitrate reductase complex (Figure 2B). The Molecular Function category revealed alterations in catalytic activity, as well as categories related to iron–sulfur cluster metabolism (Figure 2C). In the Biological Process category, several terms related to metabolic processes such as nitrate assimilation and the aromatic compound catabolic process were changed (Figure 2D).

### Pathways Downregulated in Stationary Phase Based on Transcriptome Profiling

Chemotaxis and flagellar assembly were significantly downregulated (Table 1 and Supplemental Figure S1). This includes genes encoding proteins such as methyl-accepting chemotaxis proteins (MCPs), CheA, and CheY, which are involved in chemotactic signal transduction. CheA is part of a two-component system that initiates the chemotactic signal transduction cascade.^45^ The flagellar assembly pathway includes genes such as *fliC*, which encodes flagellin, which is a crucial element for flagella formation and function. Also downregulated was *fliA* (log2-fold change −1.7), which encodes the flagellar sigma factor, a member of the σ^28^ protein family, which directs RNA polymerase to transcribe flagellar genes. Such reduction in motility is characteristic of bacteria as they transition from the motile, planktonic state to a more sedentary lifestyle as the resource demand to fuel motility becomes too high.^46^

*Escherichia coli* encodes three nitrate reductases, of which one is periplasmic and two are membrane-bound. One operon encoding a membrane-bound nitrate reductase is under control of the two-component system NarXL, which responds to nitrate and nitrite. The other is under control of RpoS, and its expression is increased in stationary phase.^47^ Such redundancy is also seen in *B. thailandensis.* We found that *BTH_I1852–1854*, which is downstream from genes encoding NarXL, was among the most downregulated genes (Table 1 and Supplemental Figure S2). By contrast, *BTH_II1249–1251*, encoding the other membrane-bound nitrate reductase, is upregulated, perhaps mediated by RpoS.

In addition, we observed the expected depletion of mRNAs encoding ribosomal proteins (log2-fold range −5.9 to −1.5; Supplemental Table S1). mRNAs encoding proteins that participate in the arginine deiminase pathway (*arcDABC; BTH_I1283–1286*) were also markedly reduced. This pathway is involved in ATP generation during anaerobic growth.^48^ Also notable was the reduction of mRNAs encoding Type III Secretion System components and effectors, mRNAs encoding enzymes involved in synthesis and export of the antibacterial 4-hydroxy-3-methyl-2-alkenylquinolines (HMAQ), NADH dehydrogenase, involved in electron transport, and mRNA representing the *suf* operon, encoding proteins involved in iron–sulfur cluster biogenesis under iron limitation or oxidative stress.^49^

### Upregulated Pathways Based on Transcriptome Profiling

Benzoate degradation was markedly upregulated in the stationary phase. Benzoate may derive from degradation of aromatic compounds and in some species phenylalanine, and the steps involved in its aerobic degradation include its conversion to catechol and further processing in the β-ketoadipate pathway (Supplemental Figure S3).^50^ Upregulated genes include *BTH_II0471–0473*, encoding benzoate dioxygenase catalyzing the first step toward conversion to catechol as well as all genes encoding enzymes required for conversion of catechol to succinyl-CoA (Table 1). Also upregulated was the operon *BTH_II0488–0491* encoding the anthranilate-inducible ortho-halobenzoate dioxygenase, which converts anthranilate to catechol;^51^ anthranilate (2-aminobenzoate) is an intermediate in the synthesis and degradation of tryptophan.^52^ Aromatic amino acids were present in the culture medium used.

Other notable upregulated gene clusters included *BTH_II0562–0574,* encoding proteins with homology to the barbamide biosynthetic enzymes from *Lyngbya majuscule* and *BTH_II0542–0552*, encoding biosynthetic enzymes for the exopolysaccharide cepacian. Biosynthesis of nucleotide sugars was also upregulated, reflecting a need for building blocks of carbohydrates and glycoconjugates. In addition, cepacian promotes biofilm formation, as do O-antigen-linked nucleotide sugars.^53,54^

### Proteome Profiling

Changes in protein levels were monitored using quantitative mass spectrometry, using the same samples used for transcriptomics, resulting in identification of 3,033 proteins of a total of 5,562 entries in UniProt (for details on the workflow, see Supplemental Figure S4). Using a log2-fold change cutoff of |0.5|, we identified 832 differentially expressed proteins (DEPs), with 552 upregulated and 280 downregulated during the stationary phase (Figure 3A-B). The principal component analysis (PCA) revealed particularly tight clustering of samples collected in the stationary phase (Figure 3C). The top 20 accumulating or depleted proteins are shown in Table 2, and the entire data set is provided in Supplemental Table S3. Several key pathways were highlighted through KEGG pathway analysis (Figure 4A and Supplemental Table S4). Expected changes included a decrease in ribosome biogenesis and an increased accumulation of proteins related to fatty acid degradation, glycolysis, and amino acid metabolism.

**Figure 3:**
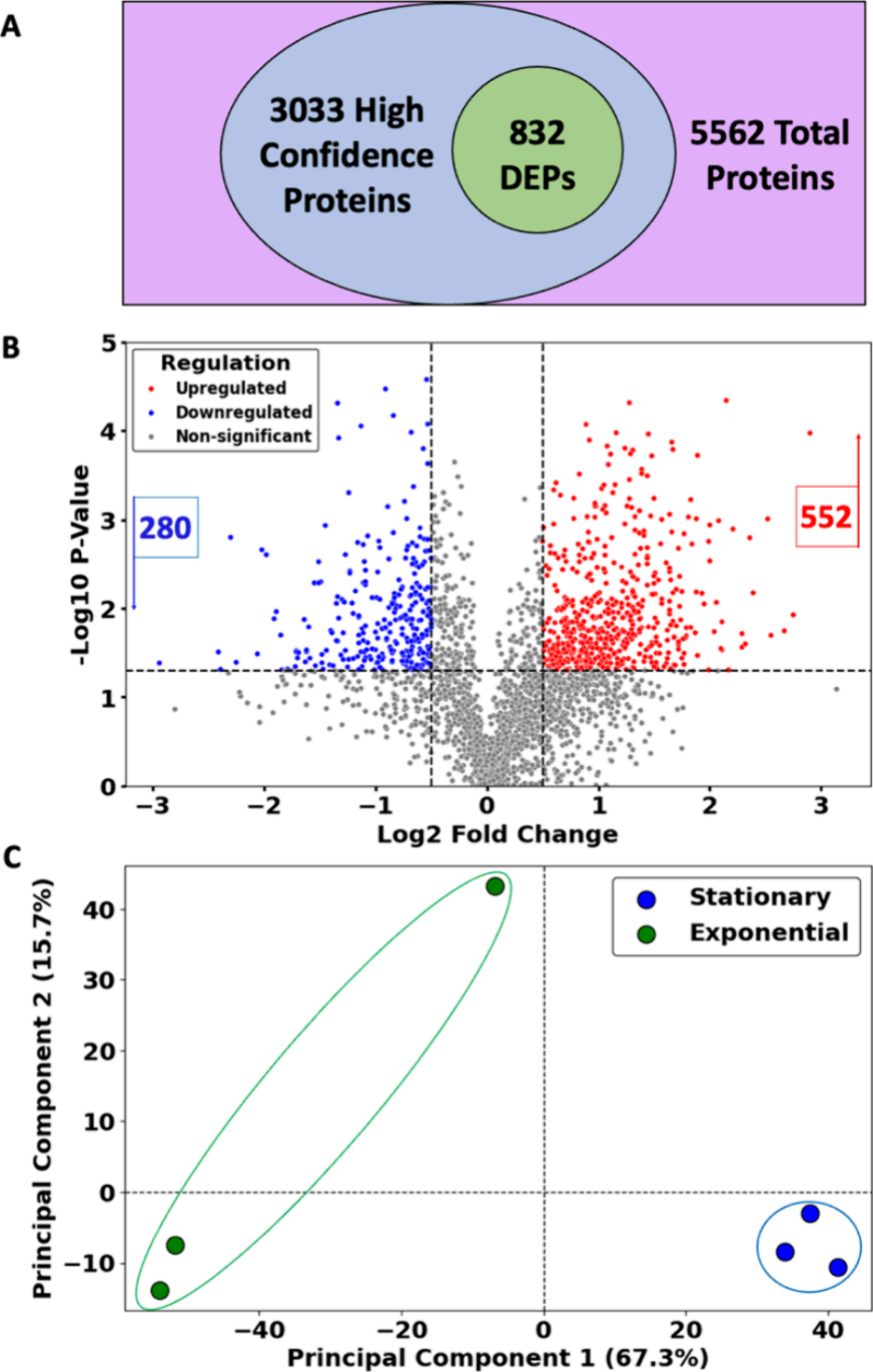
**Mass spectrometry quantitative proteomics profiling.** A. Venn diagram illustrating the overlap of differentially expressed proteins (832) relative to proteins detected with high confidence (3,033). The total number of proteins (5,562) in *B. thailandensis* is indicated. B. Volcano plot of differentially expressed proteins in stationary relative to exponential phase. The horizontal dashed line represents a p-value of 0.05, and the vertical dashed lines represent the log2-fold change of |0.5|. Upregulated proteins are colored red (552), while downregulated proteins blue (280). C. Principal Component Analysis (PCA) of proteomic profiles. Each point represents a biological replicate with colors representing the exponential phase (green) and the stationary phase (blue).

**Figure 4:**
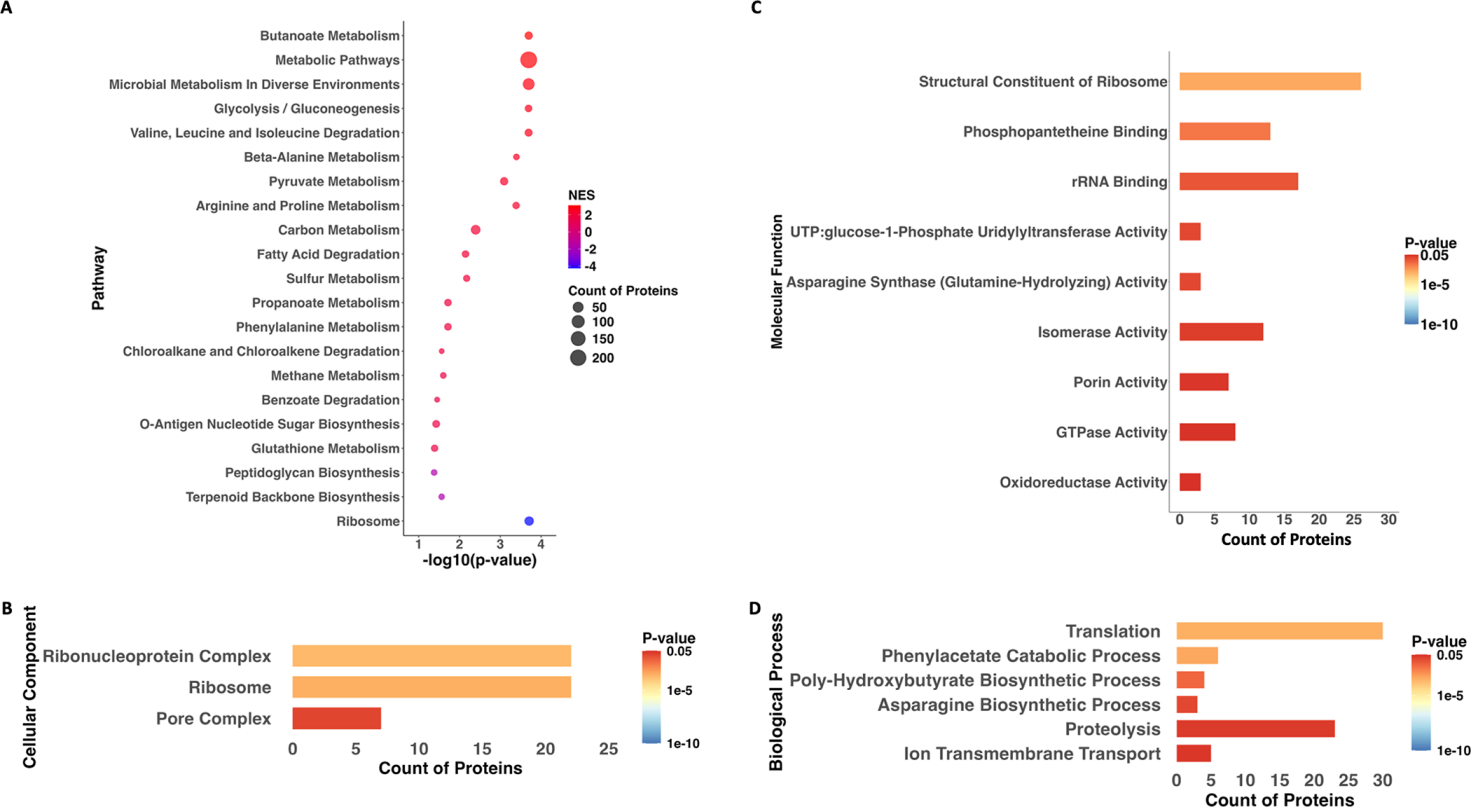
**Quantitative proteomics KEGG pathways and gene ontology.** A. Bubble plot illustrating the altered KEGG pathways during the stationary phase compared to the exponential phase. Each circle represents a pathway, with the size of the circle indicating the number of DEPs in the pathway. The *x*-axis represents the −log10­(p-value) of the change. The colors of the circles indicate the Normalized Enrichment Score (NES), with red shades representing upregulated pathways (NES closer to 2) and blue shades representing downregulated pathways (NES closer to −4). B-D. Bar plots showing the enriched Gene Ontology (GO) terms among differentially expressed proteins for Cellular Component, Molecular Function, and Biological Process, respectively. The axis X shows the observed protein count associated with each GO term. The color of each bar corresponds to the calculated p-value, with more red colors representing higher levels of enrichment significance (lower p-value) and more blue colors representing lower levels of enrichment significance (higher p-value).

**Table 2:** Top Differentially Expressed Proteins in the Stationary Phase

**Protein ID**	**Gene ID**	**Log2 Fold Change**	**Log2 Fold Change Standard Error**	**Regulation**	**Protein Name**
Q2T1 V4	BTH_I0287	2.90	0.24	Up	Streptavidin, putative
Q2SX55	BTH_I1965	2.75	0.26	Up	Acyltransferase family protein
Q2T4D5	BTH_II1770	2.67	0.32	Up	Major surface protein 3
Q2STH1	BTH_I3286	2.55	0.77	Up	Small-conductance mechanosensitive channel
Q2T5L4	BTH_II1339	2.52	0.34	Up	DUF4347 domain-containing protein
Q2T5N6	BTH_II1317	2.39	0.28	Up	GYD domain-containing protein
Q2T4C7	BTH_II1778	2.36	0.3	Up	Cytochrome c
Q2T885	BTH_II0414	2.32	0.34	Up	Osmotically inducible protein Y domain protein
Q2T7T5	BTH_II0564	2.29	0.38	Up	BarB2
Q2T7 V8	BTH_II0541	2.29	0.28	Up	Insertion element IS402-like domain-containing Protein
Q2T176	BTH_I0516	2.21	0.25	Up	Ribosomal natural product, two-chain TOMM family
Q2T4M8	BTH_II1677	2.17	0.28	Up	Phenolpthiocerol synthesis type-i polyketide synthase ppsa
Q2STN6	BTH_I3219	2.15	0.02	Up	Chitin binding protein, putative
Q2SZS0	BTH_I1025	2.11	0.34	Up	DNA-binding response regulator KdpE
Q2T2M8	BTH_I0090	2.11	0.24	Up	Periplasmic heavy metal sensor
Q2T4H3	BTH_II1732	2.10	0.40	Up	3,4-dihydroxyphenylacetate 2,3-dioxygenase
Q2T177	BTH_I0515	2.08	0.12	Up	ABC transporter ATPase
Q2T514	BTH_II1540	2.06	0.21	Up	MoaF protein
Q2T883	BTH_II0416	2.04	0.31	Up	Acetate kinase (Acetokinase)
Q2T568	BTH_II1485	2.00	0.18	Up	Transcriptional regulator, AsnC family
Q2SY43	BTH_I1618	–2.94	0.55	Down	Citrate-proton symporter
A0A1W5T746	BTH_I0643	–2.41	0.46	Down	Protein RecA
Q2SZ29	BTH_I1273	–2.39	0.47	Down	YceI-like family protein
Q2SVQ6	BTH_I2472	–2.30	0.32	Down	Ribose operon repressor RbsR PA1949
Q2T318	BTH_II2243	–2.25	0.35	Down	4-hydroxy-3-methylbut-2-enyl diphosphate reductase
Q2SZV7	BTH_I0988	–2.06	0.35	Down	Lipoprotein, putative
Q2SXE2	BTH_I1877	–2.02	0.23	Down	Iron-binding protein IscA
Q2SXE3	BTH_I1876	–1.98	0.17	Down	Iron–sulfur cluster assembly scaffold protein IscU
Q2STR0	BTH_I3195	–1.91	0.40	Down	30S ribosomal protein S21
Q2T007	BTH_I0938	–1.89	0.31	Down	DUF4399 domain-containing protein
Q2T7N4	BTH_II0615	–1.85	0.23	Down	GTP cyclohydrolase FolE2
Q2SVH2	BTH_I2560	–1.84	0.35	Down	Multidrug resistance protein
Q2SZ30	BTH_I1272	–1.79	0.38	Down	YceI-like family protein
Q2SU18	BTH_I3077	–1.72	0.31	Down	50S ribosomal protein L7/L12
Q2SXS7	BTH_I1737	–1.71	0.29	Down	Elongation factor P (EF-P)
Q2SU42	BTH_I3053	–1.71	0.26	Down	50S ribosomal protein L6
Q2SWJ7	BTH_I2181	–1.70	0.30	Down	30S ribosomal protein S18
Q2T8W4	BTH_II0183	–1.69	0.23	Down	Flagella basal body P-ring formation protein FlgA
Q2T0C9	BTH_I0814	–1.64	0.23	Down	Sulfite reductase
Q2SU26	BTH_I3069	–1.63	0.42	Down	30S ribosomal protein S10

The GO analysis reflected the expected change in the ribosome and ribonucleoprotein complexes along with the pore complex, all in the Cellular Component category (Figure 4B). The Molecular Function category also reflected changes in these categories (structural constituent of ribosome and rRNA binding). Other important molecular functions included oxidoreductase activity and phosphopantetheine binding (Figure 4C). In the Biological Process category, the most altered terms included translation and proteolysis (Figure 4D).

### Downregulated Proteins

The ribosome biogenesis pathway, one of the most energy-consuming biological processes,^12^ was significantly downregulated during the stationary phase (Supplemental Figure S5). Ribosomal proteins were among the downregulated DEPs, along with several proteins required for translation, such as elongation factors G and P and peptide chain release factor 2. FtsZ, required for cell division, was also down, and so was the bifunctional protein PurH, which catalyzes two steps in purine *de novo* biosynthesis.^55^ Also down was the peptidoglycan biosynthesis pathway, including proteins such as peptidoglycan D,D-transpeptidase MrdA and penicillin-binding protein, all reflecting the reduced growth and the need to conserve resources. We also noted marked downregulation of the house-keeping iron–sulfur cluster assembly proteins IscS, IscU, and IscA (Table 2).

### Upregulated Proteins

KEGG pathways classified as metabolic pathways and microbial metabolism in diverse environments were among the most markedly upregulated in the stationary phase (Figure 4A). Metabolism of fatty acids was increased, as expected. Metabolism of butanoate was significantly upregulated, including upregulation of polyhydroxybutyrate depolymerase; polyhydroxybutyrate serves as a storage form of carbon. In addition, beta-hydroxybutyrate is one of the ketone bodies that accumulate during fatty acid degradation when there is insufficient oxaloacetate for entry of acetyl-CoA into the citric acid cycle (Supplemental Figure S6).^56^ Metabolism of phenylalanine and tryptophan (Figure 4A and Supplemental Table S4) was upregulated; such upregulation is consistent with the observation that genes encoding proteins involved in degradation of benzoate and anthranilate were upregulated.^52^ Proteins involved in the biosynthesis of several secondary metabolites were also seen to accumulate, including proteins related to the biosynthesis of barbamide, malleilactone, and thailandamide.

The glutathione metabolism pathway was significantly upregulated, with key proteins such as glutathione S-transferase involved in detoxification processes to cope with increased oxidative stress (Figure 4A).^57^ Consistent with an oxidative stress response, we also noted accumulation of alkyl hydroperoxide reductase and *trans*-aconitate methyltransferase; the latter has a role in preventing accumulation of *trans*-aconitate, which is generated when the iron–sulfur cluster of the citric acid cycle enzyme aconitase is damaged by oxidative stress.^58^ DpsA (DNA protection during stress) was also upregulated, as expected (Supplemental Table S2).

The nitrate reductase encoded by *BTH_II1249–1251* was seen to accumulate, consistent with the upregulation of the corresponding genes. There was also significant accumulation of a xanthine dehydrogenase subunit; this enzyme functions in purine salvage where it promotes conversion of ATP to GTP. This in turn favors synthesis of the alarmone (p)­ppGpp.^59^ Accumulation of a diguanylate cyclase, which generates the second messenger cyclic di-GMP (c-di-GMP), was also noted (Supplemental Table S2). In most bacterial species, including *B. thailandensis*, elevated levels of c-di-GMP are associated with reduced motility and increased biofilm formation.^60^

### Protein–Protein Interaction Networks (PPI) During the Stationary Phase

A predicted protein–protein interaction (PPI) network was constructed, focusing on upregulated proteins identified by mass spectrometry. This approach was employed to pinpoint proteins that may be actively engaged in the bacterial response to stationary phase conditions. The resulting PPI network consisted of 552 nodes (proteins) and 839 edges (interactions), with an average node degree of 3.04 and a maximum degree of 26. Notably, the observed number of edges (839) significantly exceeded the expected number (587), as evidenced by a PPI enrichment p-value of <1.0 × 10^–16^. This enrichment suggests a highly interconnected network indicative of a robust and coordinated system that surpasses random chance.

Functional enrichment analysis revealed stationary phase PPIs associated with metabolic processes, such as fatty acid and amino acid metabolism, reflecting the metabolic reprogramming necessary for survival under nutrient-limited conditions (Figure 5). Corresponding KEGG pathways and GO processes are detailed in Supplemental Figure S7, with topological analysis results of each node provided in Supplemental Table S5.

**Figure 5:**
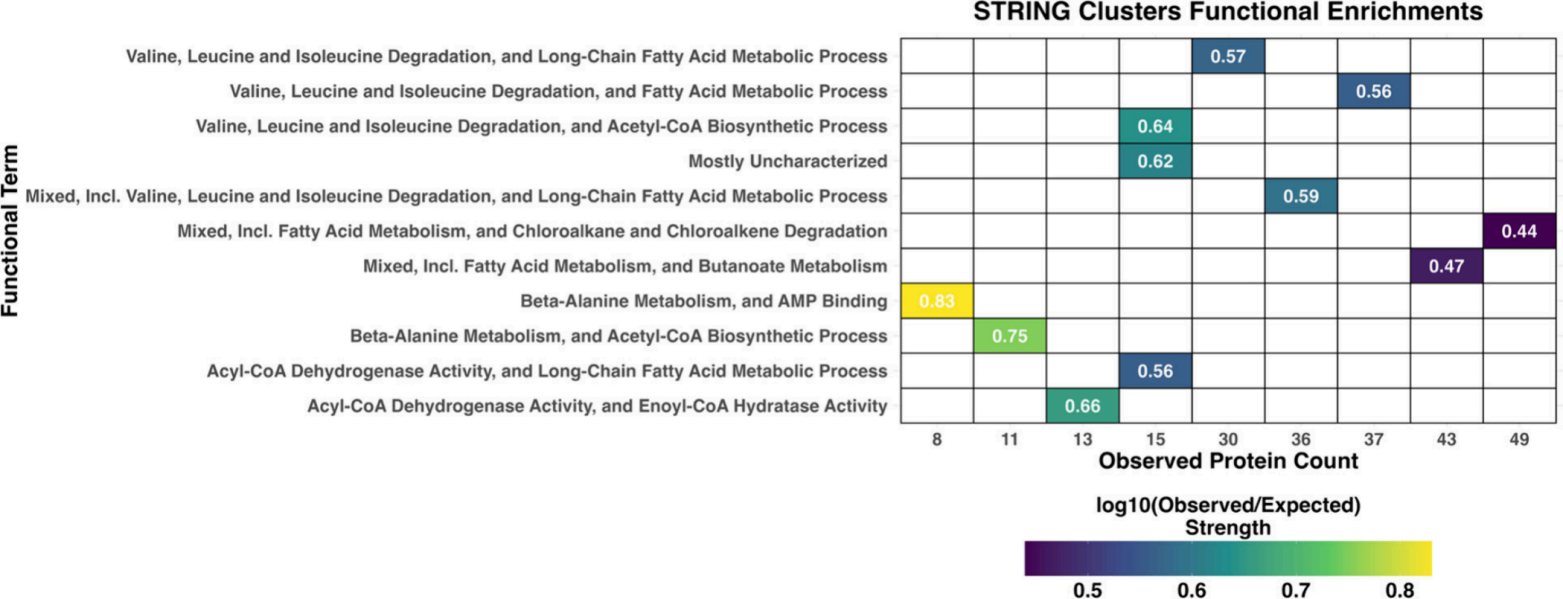
**Protein–protein interaction (PPI) network and functional enrichment.** Heatmap displaying the enrichment strength of STRING clusters based on the observed gene count for each functional term description. The color intensity represents the log10 of the observed to expected ratio, with darker colors signifying stronger interactions.

Formate Dehydrogenase (FDH) beta subunit (Q2T7E2) was identified as the hub protein with the most (26) interacting partners (Table 3). FDH is integral to the microbial metabolism of one-carbon compounds, particularly within the formate oxidation pathway, which is essential for maintaining redox balance under nutrient-limited conditions.^61^ The interaction of FDH with enzymes such as adenylylsulfate kinase and cysteine synthase suggests a potential linkage between formate metabolism and sulfur assimilation, which is crucial for synthesizing sulfur-containing amino acids and cofactors. Additionally, interactions with malate synthase and phosphoenolpyruvate carboxykinase suggest FDH’s involvement in coordinating the glyoxylate cycle and gluconeogenesis, thereby reorganizing carbon flux during periods of nutrient scarcity. The network further reveals interactions between FDH and nitrate reductase subunits, suggesting its association with the utilization of alternative electron acceptors under the oxygen-limited conditions typical of the stationary phase. Furthermore, interactions with proteins associated with aromatic compound degradation could provide an alternative carbon source under nutrient-limited conditions.

**Table 3:** Main Hub Proteins^a^

**Hub Protein**	**Protein Annotation**	**Number of Interactions**
Q2T7E2	Formate dehydrogenase, beta subunit	26
Q2T882	Phosphate acetyl butyryltransferase family protein	24
Q2STF6	Bifunctional protein PutA	22
Q2T4A5	3-hydroxyisobutyrate dehydrogenase (HIBADH)	22
Q2T066	Aldehyde dehydrogenase family protein	21
Q2T2J3	Acyl-CoA dehydrogenase	21
Q2SWN6	Fatty oxidation complex, alpha subunit, putative	20
Q2T4A2	Acyl-CoA dehydrogenase	20
Q2T4A3	AMP-binding enzyme	19
Q2SUJ0	Phenylacetate degradation probable enoyl-CoA hydratase paaB	17
Q2SWA7	Aldehyde dehydrogenase	17
Q2T125	Enoyl-CoA hydratase/isomerase family protein	16
Q2T883	Acetate kinase (acetokinase)	14
Q2SZL3	Acyl-CoA dehydrogenase domain protein	14
Q2T4A4	Methylmalonate-semialdehyde dehydrogenase (CoA acylating)	14
Q2T6Q3	Enoyl-CoA hydratase/isomerase family protein	14
Q2T3K0	Malate synthase G	13
Q2SYR3	Glutaryl-CoA dehydrogenase (ETF)	13
Q2STC4	Methylmalonate-semialdehyde dehydrogenase (CoA acylating)	13
Q2T1J8	Fatty oxidation complex, alpha subunit, putative	13
Q2T4A6	Enoyl-CoA hydratase/isomerase family protein	13
Q2SX31	Malate synthase	12
Q2SWC1	Acetoacetyl-CoA reductase	12
Q2T7 × 9	Acyl-CoA dehydrogenase domain protein	12

a The UniProt accession numbers for the main hub proteins are shown, along with a functional description and the total number of interacting proteins.

Phosphate acetyl/butyryltransferase family protein (Q2T882) was identified as another critical hub within the PPI network, interacting with 25 other proteins. Interacting proteins with roles in fatty acid β-oxidation, branched-chain amino acid metabolism, and proteins involved in energy storage and mobilization via polyhydroxyalkanoates (PHAs), which is essential for survival during prolonged stationary phase conditions, speak to the integration of distinct metabolic pathways. Connections with malate synthase and isocitrate lyase further emphasize the rerouting of carbon metabolism through the glyoxylate cycle.

The network analysis generally highlights significant clusters involved in fatty acid metabolism and amino acid degradation such as the cluster related to valine, leucine, and isoleucine degradation (Figure 5). Taken together, these findings indicate a strategic reallocation of resources to maintain metabolic flexibility and cellular integrity under nutrient-limited conditions.

### Integrative Analysis of RNA-Seq and Quantitative Proteomics Data

To gain a comprehensive understanding of the molecular changes in *B. thailandensis* during the stationary phase, we performed an integrative analysis of RNA-Seq and quantitative proteomics data. This analysis aimed to correlate mRNA levels with protein abundance and identify shared and divergent regulatory mechanisms at the transcription and translational levels. We first assessed the correlation between RNA-Seq and proteomics data by comparing the normalized log2-fold changes in differentially expressed genes and proteins. A scatter plot was used to visualize this relationship (Figure 6A), with a Pearson correlation coefficient (r = 0.4) indicating a moderate positive correlation. This moderate correlation is consistent with previous studies, which have shown that mRNA and protein levels do not always correlate strongly.^62,63^

**Figure 6:**
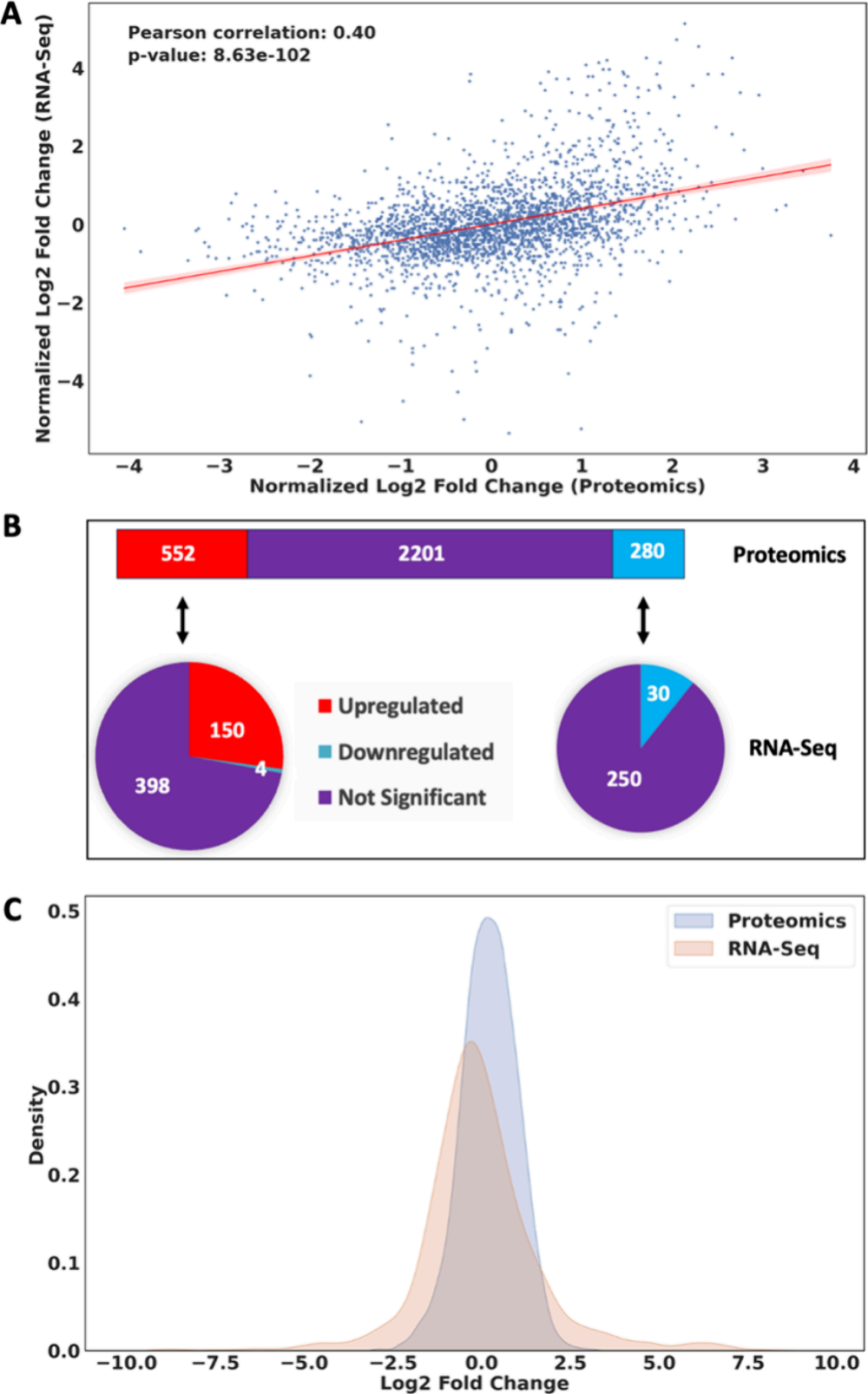
**Correlation of RNA-seq and quantitative proteomics data.** A. Correlation between proteomics and RNA-seq data, with a Pearson correlation coefficient of 0.4 and a p-value of 8.63 × 10^–102^. The *x*-axis represents the normalized log2-fold change in proteomics, while the *y*-axis represents the normalized log2-fold change in RNA-seq data. Each point represents a protein/gene pair, and the red fitted regression line illustrates the trend. B. The distribution of differentially expressed proteins and their corresponding gene expression changes. The top bar diagram shows downregulated (blue) and upregulated (red) proteins. The pie charts reflect the proportion of up-regulated (left) and down-regulated (right) proteins that are up-regulated (red) or down-regulated (blue) in the RNA-seq data. C. Density plot of log2-fold changes from proteomics (blue) and RNA-seq (orange) data. The *x*-axis represents the log2-fold change, and the *y*-axis represents the density of the data points.

We also compared the number of differentially expressed genes and proteins. From the 552 upregulated proteins identified in the proteomics data, 150 were also upregulated in the RNA-Seq data, while 4 were downregulated and 398 were not significant (given the applied cutoffs). Similarly, of the 280 downregulated proteins, 30 were also downregulated in the RNA-Seq data, while 250 were not significant (Figure 6B). Overall, we found that 180 proteins had the same expression trend in both data sets, whereas only 4 proteins exhibited inverse regulation (Supplemental Table S6). Two pathways were upregulated in both the RNA-Seq and the proteomics data: benzoate degradation and O-antigen nucleotide sugar biosynthesis.

The distribution of log2-fold changes was compared between RNA-Seq and proteomics data using a density plot (Figure 6C). The distribution of log2-fold changes in RNA-Seq data was broader and of lower amplitude, while the proteomics data showed a narrower and taller distribution (the corresponding distributions are visualized by a violin plot in Supplemental Figure S8). This indicates that the wider range of expression changes seen at the mRNA levels is not reflected at the protein level. This difference speaks to marked post-transcriptional regulation.

### Regulation of RpoS

RpoS is thought to have arisen from an RpoD duplication event prior to the emergence of Proteobacteria and is conserved in proteobacterial species; however, RpoS regulons are not well-conserved between species.^64^ While RpoS levels are low in the exponential phase, they increase markedly in the stationary phase due to a combination of regulatory events.^9^ Such a pattern was not observed in *B. thailandensis*. In the RNA-seq data set, *rpoS* mRNA levels did not meet the filtration criteria for significant differential expression, with a log2-fold change of only 1.2. Furthermore, proteomics data also indicated that RpoS was not markedly increased (log2-fold change of 1.08). To further investigate these findings, we performed RT-qPCR analysis to examine the temporal expression of *rpoS*. A transient but modest increase in RpoS mRNA levels was observed during the initial entry into the stationary phase, followed by a return to initial levels (Figure 7A). By comparison, using *lacZ* fused to the *B. pseudomallei rpoS* promoter, a significant increase was observed on entry into the stationary phase, followed by a modest decrease.^65^

**Figure 7:**
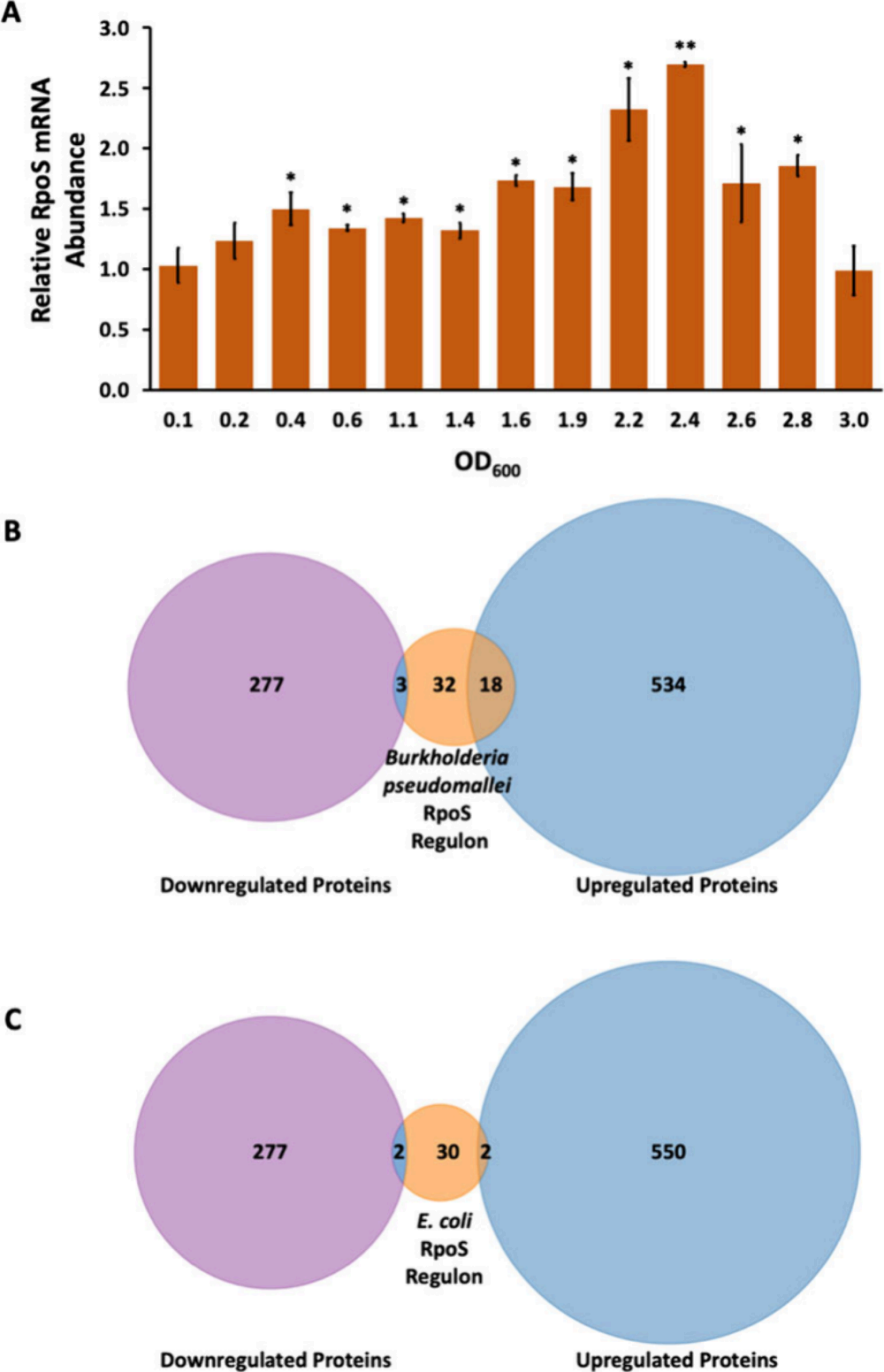
**Regulation of RpoS.** A. RT-qPCR analysis of the *rpoS* mRNA levels. Relative mRNA abundance is shown as a function of OD_600_, normalized for the cell count. Error bars represent the standard deviation from three experiments. Asterisks reflect statistically significant differences compared to the mRNA abundance at OD_600_ = 0.1 based on a Student’s *t* test; *, *p* < 0.05; **, *p* < 0.001. B. Venn diagram comparing differentially expressed proteins regulated by RpoS in *B. pseudomallei* (orange) with differentially expressed proteins in *B. thailandensis* stationary phase; upregulated proteins are shown in blue, and downregulated proteins are shown in purple. C. Venn diagram illustrating the intersection of differentially expressed proteins regulated by RpoS in *E. coli* (orange) with differentially expressed proteins in *B. thailandensis* stationary phase; upregulated proteins are shown in blue and downregulated proteins in purple.

We examined the RpoS regulon in *Burkholderia pseudomallei*, determined based on mass spectrometric identification of proteins accumulating in an *rpoS* mutant strain.^42^ By comparison to proteins differentially expressed in the *B. thailandensis* stationary phase, we identified an only modest overlap (Figure 7B). For example, proteins such as polyphosphate kinase 2 (Ppk2), carboxymuconolactone decarboxylase (PcaC), phasin (PhaP), and a nonribosomally encoded peptide/polyketide synthase (CmaB) were upregulated in *B. thailandensis* during the stationary phase, and they were part of the *B. pseudomallei* RpoS regulon (Supplemental Table S7).

We also compared our findings with the *E. coli* RpoS regulon, likewise identified based on identification of accumulating proteins in an *rpoS* mutant strain.^43^ The overlap was minimal, suggesting distinct regulatory mechanisms between these species (Figure 7C). For instance, while *E. coli* RpoS positively regulates the gene encoding GroES (10 kDa chaperonin), it was downregulated in the *B. thailandensis* stationary phase (Supplemental Table S8). Taken together, our findings suggest that *B. thailandensis* employs unique regulatory mechanisms for RpoS that do not result in its marked accumulation during the stationary phase.

## Discussion

The transition to the stationary phase represents a crucial adaptive strategy that enables bacteria to endure environmental stress, nutrient scarcity, and other harsh conditions.^7,66^ Such adverse conditions are likely the norm rather than the exception, and they may, for instance, characterize a host environment rife with antibacterial defenses. For these reasons, the physiology and metabolism of stationary phase cells has been characterized in many species,^7,66^ leading to the identification of several general features. Consistent with such expectations, we found that *B. thailandensis* undergoes extensive metabolic reprogramming, including a shift toward energy conservation, the activation of alternative metabolic pathways, and reduced ribosome biogenesis.^8^ However, our analyses also uncovered unusual regulatory adaptations, for instance, involving the sigma factor RpoS.

Integrating transcriptomic and proteomic data has the advantage of providing insights into the molecular mechanisms that underlie the transition to the stationary phase, beyond the proteome alterations that arise on account of changes in mRNA abundance. As previously reported,^17^ we also found an only limited correspondence between mRNA and protein abundance (Figure 6). Such a modest correlation speaks to post-transcriptional regulation playing a marked role in determining protein abundance. These events may include regulation at the level of translation, perhaps with a contribution from small noncoding RNAs (sRNAs).^67^ In *Burkholderia cepacia*, for instance, a number of sRNAs have been characterized and shown to interact with target mRNAs, assisted by RNA chaperones such as Hfq; such interactions may, for example, lead to occlusion of the ribosome binding site and impaired initiation of translation.^68^ Protein stability may also be regulated, perhaps directed by post-translational modifications.^69^

### Energy-Saving Measures

*B. thailandensis* significantly downregulates nitrogen metabolism, motility, and ribosome biogenesis during the stationary phase, mirroring similar adaptations in other bacteria.^7,66^ Our proteomic analysis identified a marked reduction in core ribosomal proteins and elongation factors, reinforcing the tenet that minimizing ribosome biogenesis is a universal strategy among bacteria during nutrient-limited conditions to conserve energy.^12^

Additionally, the repression of motility-related pathways, such as flagellar assembly and chemotaxis, reflects the expected strategic shift from a motile to a sessile lifestyle, prioritizing biofilm formation as a survival mechanism under nutrient stress.^46^ This energy-saving response is observed across various species, as flagellar gene repression reduces the energetic cost of motility. Several gamma-proteobacterial species have even been shown to actively disassemble their flagellar filaments when nutrients are scarce, effectively conserving energy. This programmed loss of flagella leaves behind only what was termed a “relic” of the ejected flagellar motor, associated with a protein complex thought to prevent periplasmic leakage.^70^

Consistent with the goal of reducing the energetic cost of motility, we observed a reduced accumulation of *fliA* mRNA, encoding the flagellar sigma factor required for transcription of flagellar genes. In *E. coli* and related bacteria, *flhDC*, which encodes the transcriptional master regulator FlhDC, is regulated at several levels; FlhDC in turn stimulates transcription of numerous genes, including *fliA*.^71^ We observe a modest reduction of *flhDC* mRNA, consistent with a comparable regulatory mechanism in *B. thailandensis* (FlhDC was not confidently detected in the proteome analysis). FlhDC was also reported to regulate flagellar genes in the rice pathogen *Burkholderia glumae*.^72^ Since FliA has also been implicated in driving transcription of chemotaxis-related genes, the reduced accumulation of *fliA* mRNA (and the associated 0.6-fold protein abundance in stationary phase; Supplemental Table S3) likely contributes to the observed repression of motility and chemotaxis pathways.

### Metabolic Changes

In the context of nitrogen metabolism, we observed the differential accumulation of mRNA corresponding to two nitrate reductase operons. *BTH_I1852–1854* is expected to be under control of nitrate and nitrite, and the corresponding mRNA was among the most depleted in the stationary phase, whereas mRNA corresponding to *BTH_II1249–1251* was increased (Table 1 and Supplemental Figure S2). While regulation of nitrogen metabolism in stationary phase is variable depending on both species and specific environments,^47^ upregulation of a membrane-bound nitrate reductase was seen in *Pseudomonas aeruginosa* exposed to the low oxygen environment of the cystic fibrosis lung.^73^ This would be equivalent to the oxygen limitation experienced during the stationary phase due to high cell density and limited diffusion. Since nitrate may be used as an alternate electron acceptor when oxygen is limiting,^47^ we speculate that this might lead to its depletion in stationary phase, rationalizing the downregulation of the nitrate reductase encoded by *BTH_I1852–1854*.

Interestingly, *B. thailandensis* upregulates pathways responsible for degradation of the aromatic carbon sources benzoate and anthranilate (Table 1 and Supplemental Figure S3). Both compounds are converted to catechol,^52^ which enters the β-ketoadipate pathway and is converted to intermediates in the citric acid cycle, suggesting a strategic redirection of metabolic flux toward alternative carbon sources when preferred nutrients are limited. In *P. aeruginosa*, anthranilate has been shown to accumulate transiently in the stationary phase, and this leads to induction of the operon encoding the dioxygenase, which converts anthranilate to catechol.^74^ Anthranilate is an intermediate in the catabolism of tryptophan by the aerobic kynurenine pathway,^75^ indicating a possible source of anthranilate.

Notably, anthranilate was shown to regulate pathogenicity-related phenotypes in *P. aeruginosa* such as biofilm formation and antibiotic sensitivity, suggesting a need for careful regulation of its cellular levels.^74^ We also note that anthranilate is an intermediate in the synthesis of 4-hydroxy-3-methyl-2-alkenylquinolines (HMAQs), which are analogous to the *Pseudomonas* quinolone signal PQS, although they do not appear to have comparable roles in quorum sensing;^76^ the increase in anthranilate catabolism correlates with a reduction in the mRNA encoding all of the enzymes involved in the conversion of anthranilate to HMAQ (*BTH_II1929–1935*; log2-fold change of −4.6 to −2.9; Supplemental Table S1). While all of the corresponding proteins were detected, no statistically significant change in abundance was observed in the stationary phase (Supplemental Table S3).

The observed upregulation of alternative energy pathways such as fatty acid degradation further exemplifies the metabolic flexibility of *B. thailandensis*. This adaptive strategy is reminiscent of metabolic shifts seen in other bacteria.^13^ Fatty acids released from the degradation of membrane lipids are scavenged and converted to acyl-CoA, which may then be degraded by the process of β-oxidation. Phosphate acetyl/butyryltransferase was predicted as a critical hub in the PPI network, linked to acetyl-CoA production, the glyoxylate cycle, and fatty acid degradation. Its interactions with proteins involved in polyhydroxyalkanoate (PHA) metabolism further highlight the importance of energy storage and mobilization during the stationary phase.

Our findings also indicate a significant induction of glutathione metabolism, reflecting the expected response to oxidative stress during the stationary phase. The increase in glutathione S-transferase and related enzymes (Supplemental Table S4) suggests a targeted strategy to mitigate reactive oxygen species (ROS), a common challenge under nutrient limitation.^77^

The increase in ROS during the stationary phase may also impact iron–sulfur cluster proteins, which are used for a range of essential cellular functions. These clusters are very sensitive to oxidative stress, which can lead to their destabilization or degradation. The stress-responsive Suf (sulfur mobilization) and the house-keeping Isc (iron–sulfur cluster) systems are responsible for iron–sulfur cluster biogenesis and repair.^78^ In *E. coli*, expression of the Isc machinery is regulated by iron availability, but not by oxidative stress, although oxidative stress was shown to inactivate the Isc system.^79^ That Isc protein levels are maintained despite this inactivation was inferred to suggest an alternate function. By contrast, *suf* genes are upregulated in response to oxidative stress. We observed a significant depletion of the IscSUA proteins in the stationary phase (Table 2), a depletion that to our knowledge has not been previously reported in other bacterial species. Curiously, the expression of the *iscSUA* operon was increased ∼ 2-fold (Supplemental Table S1), indicating regulation of protein abundance post-transcriptionally. By contrast, *suf* genes were significantly repressed (Supplemental Table S1), although protein abundance was only modestly reduced (Supplemental Table S3). Our data suggest that the metabolic priorities in the stationary phase include a reduced need for iron–sulfur cluster biogenesis.

### RpoS

One of the striking findings of this analysis is that RpoS levels only modestly increased during the stationary phase. RpoS is found only in Proteobacteria, and genes have likely been recruited to the RpoS regulons through different selective pressures. As a consequence, RpoS regulons vary between species.^9,64^ In *E. coli*, the cellular concentration of RpoS was found to increase several fold in stationary phase due to a combination of very complex regulatory events.^80^ Such increase favors RpoS as it competes with the housekeeping sigma factor, σ,^70^ for binding to RNA polymerase.^11^ Some of these regulatory events, including transcriptional control and the function of proteases in RpoS degradation during exponential phase, were also reported in *P. aeruginosa*.^81^ In other bacterial species, including *B. pseudomallei*, a marked increase in *rpoS* expression has likewise been reported on entry into stationary phase.^65^ The apparent divergence from the canonical regulation of RpoS observed in species such as *E. coli* and *B. pseudomallei* is intriguing and suggests a possible evolution of RpoS-independent regulatory networks in *B. thailandensis* or RpoS-mediated regulation of gene expression that is not dependent on marked changes in protein levels.

RpoS bypass mechanisms are not without precedent, and some bacteria even lack RpoS homologues. For instance, the foodborne pathogen *Campylobacter jejuni* lacks stress-related sigma factors such as RpoS, and it relies instead on RpoN (σ^54^).^82^ In other species, such as *Vibrio cholerae*, sigma factor RpoE is important for adaptation to stationary phase along with RpoS, such that RpoS is essential for a general stress response, whereas RpoE is more specialized and crucial for responding to envelope stress and maintaining cell viability.^83^ In *B. pseudomallei*, RpoE has been associated with regulation of genes linked to processes such as stress responses and metabolic pathways,^84^ suggesting that *B. thailandensis* RpoE may likewise participate in effecting differential gene expression in stationary phase. RpoN, named for its role in nitrogen assimilation and metabolism in *E. coli*, has been implicated in regulation of genes associated with a range of processes, from flagellar motility to O-antigen expression in various bacterial species.^85^ *Burkholderia* species encode two RpoN homologues, which have been linked to intracellular survival and regulation of type III secretion system genes.^86^ These considerations point to a possible role for *B. thailandensis* RpoE and RpoN in differential gene expression in the stationary phase. It is also conceivable that secondary messengers such as (p)­ppGpp, which accumulates during the stringent response imposed by nutrient limitation, affect the ability of alternate sigma factors such as RpoS to associate with core RNA polymerase, without depending on significant changes in protein levels, thus resulting in differential gene expression patterns.^12^

Moreover, it is plausible that *B. thailandensis* employs post-translational modifications, such as phosphorylation, acetylation, or glycosylation, to modulate the activity of either RpoS, other regulatory proteins, or even RNA polymerase subunits to favor the association of core RNA polymerase with alternate sigma factors. For instance, tyrosine kinases have been described in *B. cenocepacia* and reported to be involved in growth under nutrient limitation.^87^ Similarly, loss of O-linked protein glycosylation results in global proteome changes.^88^ Exploring the extent of post-translational modifications in *B. thailandensis* could unveil novel aspects of bacterial stress adaptation.

## Conclusions

Taken together, this analysis provides a detailed picture of the molecular changes that occur in *B. thailandensis* during the transition from the exponential to stationary phase. The coordinated downregulation of energy-intensive processes, such as nitrogen metabolism and motility, along with the upregulation of alternative carbon source metabolism and stress response pathways, reflects the strategic resource reallocation to ensure survival under nutrient-depleted conditions. The modest change in RpoS protein abundance suggests that *B. thailandensis* may rely on unique regulatory approaches. Notably, the only modest correlation between changes in mRNA and protein abundance suggests a need to investigate post-transcriptional regulation, such as roles for sRNA in translation initiation and functional consequences of post-translational modification.

These insights into metabolic adaptation and regulatory flexibility provide a foundational understanding of microbial physiology and open new perspectives for biotechnological exploitation. Our findings open several avenues for future research, particularly into alternative regulatory networks in *B. thailandensis*, that may bypass traditional RpoS control. Moreover, the metabolic flexibility observed in *B. thailandensis* suggests potential biotechnological applications, especially in bioremediation. The ability to degrade aromatic compounds could be harnessed to degrade environmental pollutants, making it a promising candidate for cleaning contaminated soils and water systems.

## Supplementary Material













## Data Availability

The mass spectrometry proteomics data have been deposited to the ProteomeXchange Consortium via the PRIDE^89^ partner repository with the data set identifier PXD056625 and 10.6019/PXD056625. The RNA-seq data discussed in this publication have been deposited in NCBI’s Gene Expression Omnibus^90^ and are accessible through GEO Series accession number GSE 279483 (https://www.ncbi.nlm.nih.gov/geo/query/acc.cgi?acc=GSE279483). All scripts related to data integration and statistical analyses have been shared to a GitHub repository (https://github.com/Ahmed-Tohamy/rna_proteomics_integration).

## Notes

Figure S1. KEGG pathways for chemotaxis and flagellar motility. Figure S2. KEGG pathways for nitrogen metabolism. Figure S3. KEGG pathway for benzoate degradation. Figure S4. Mass spectrometry quantitative proteomics workflow. Figure S5. KEGG pathway for ribosomal proteins. Figure S6. KEGG pathway for butanoate metabolism. Figure S7. Protein–protein interaction (PPI) network functional clustering and GO enrichment. Figure S8. Violin plots for DEGs and DEPs. Table S6. Inverse expression pattern of genes and proteins. Table S7. Overlap between DEPs and *B. pseudomallei* RpoS regulon. Table S8. Overlap between DEPs and *E. coli* RpoS regulon. Table S9. RT-qPCR primer sequences. Detailed procedures for TMT labeling and protein fractionation. Detailed procedures for statistical analyses (PDF)
Table S1. DEGs (XLSX)
Table S2. KEGG pathway lists based on DEGs (XLSX)
Table S3. DEPs (XLSX)
Table S4. KEGG pathway lists based on DEPs (XLSX)
Table S5. PPI networks (XLSX)

## Abbreviations

DEGs: Differentially Expressed Genes
DEPs: Differentially Expressed Proteins
FDR: False Discovery Rate
GO: Gene Ontology
KEGG: Kyoto Encyclopedia of Genes and Genomes
NES: Normalized Enrichment Score
PPI: Protein–Protein Interaction
qPCR: Quantitative Polymerase Chain Reaction
RNA-Seq: RNA Sequencing
TMT: Tandem Mass Tag
