# Supplementary material for: Transcriptome-Proteome Profiling in Burkholderia thailandensis during the Transition from Exponential to Stationary Phase
Source: J Proteome Res. 2025 Jul 18;24(8):4082–97. doi: 10.1021/acs.jproteome.5c00223 (PMC12322963; doi:10.1021/acs.jproteome.5c00223)
Supplement: Supplementary file 1 [file pr5c00223_si_001.pdf]

Transcriptome-proteome profiling in *Burkholderia thailandensis* during the transition from exponential to stationary phase

*Ahmed Al-Tohamy,[1,2] Fabrizio Donnarumma,[3] Anne Grove[1]**

[1] Department of Biological Sciences, Louisiana State University, Baton Rouge, LA 70803, USA
[2] Department of Cell Biology, Biotechnology Research Institute, National Research Centre, Dokki, Cairo 12622, Egypt
[3] Department of Chemistry, Louisiana State University, Baton Rouge, LA 70803, USA

Contents:

Supplemental Figures:

Figure S1. KEGG pathways for chemotaxis and flagellar motility.
Figure S2. KEGG pathways for nitrogen metabolism.
Figure S3. KEGG pathway for benzoate degradation.
Figure S4. Mass spectrometry quantitative proteomics workflow.
Figure S5. KEGG pathway for ribosomal proteins.
Figure S6. KEGG pathway for butanoate metabolism.
Figure S7. Protein-protein interaction (PPI) network functional clustering and GO enrichment.
Figure S8. Violin plots for DEGs and DEPs.

Supplemental Tables:

Table S1. DEGs (Excel file).
Table S2. KEGG pathway lists based on DEGs (Excel file).
Table S3. DEPs (Excel file).
Table S4. KEGG pathway lists based on DEPs (Excel file).
Table S5. PPI networks (Excel file).
Table S6. Inverse expression pattern of genes and proteins.
Table S7. Overlap between DEPs and *B. pseudomallei* RpoS regulon.
Table S8. Overlap between DEPs and *E. coli* RpoS regulon.
Table S9. RT-qPCR primer sequences.

Detailed procedures for TMT labeling and protein fractionation

Detailed procedures for statistical analyses

Supplemental Figures:

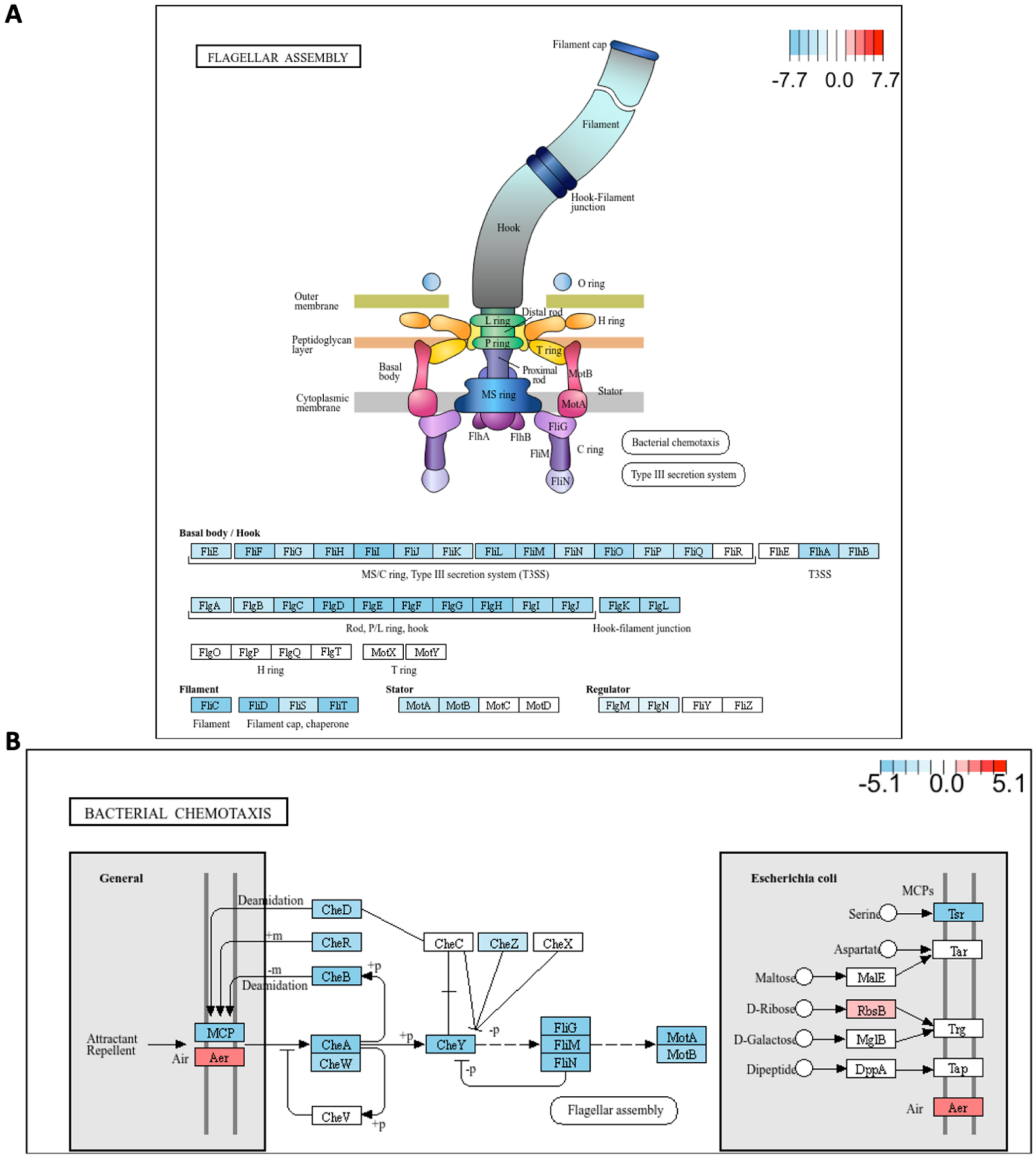

Figure S1. KEGG pathways for chemotaxis and flagellar motility. A. Schematic of the bacterial flagellar structure, indicating the arrangement of its main components (top). KEGG pathways for *B. thailandensis* flagellar motility (bottom); see https://www.genome.jp/pathway/bte02040 for identification of locus tags. B. KEGG pathways for bacterial chemotaxis; see https://www.genome.jp/pathway/bte02030 for identification of locus tags. The connection between gene products (rectangles) and chemical compounds (circles) are indicated. DEGs that meet the log2-fold change of |2| are shown in color. The color-coded scales in the top-right corners represent the log2-fold changes in gene expression in stationary phase, with shades of red reflecting upregulation and shades of blue representing downregulation.

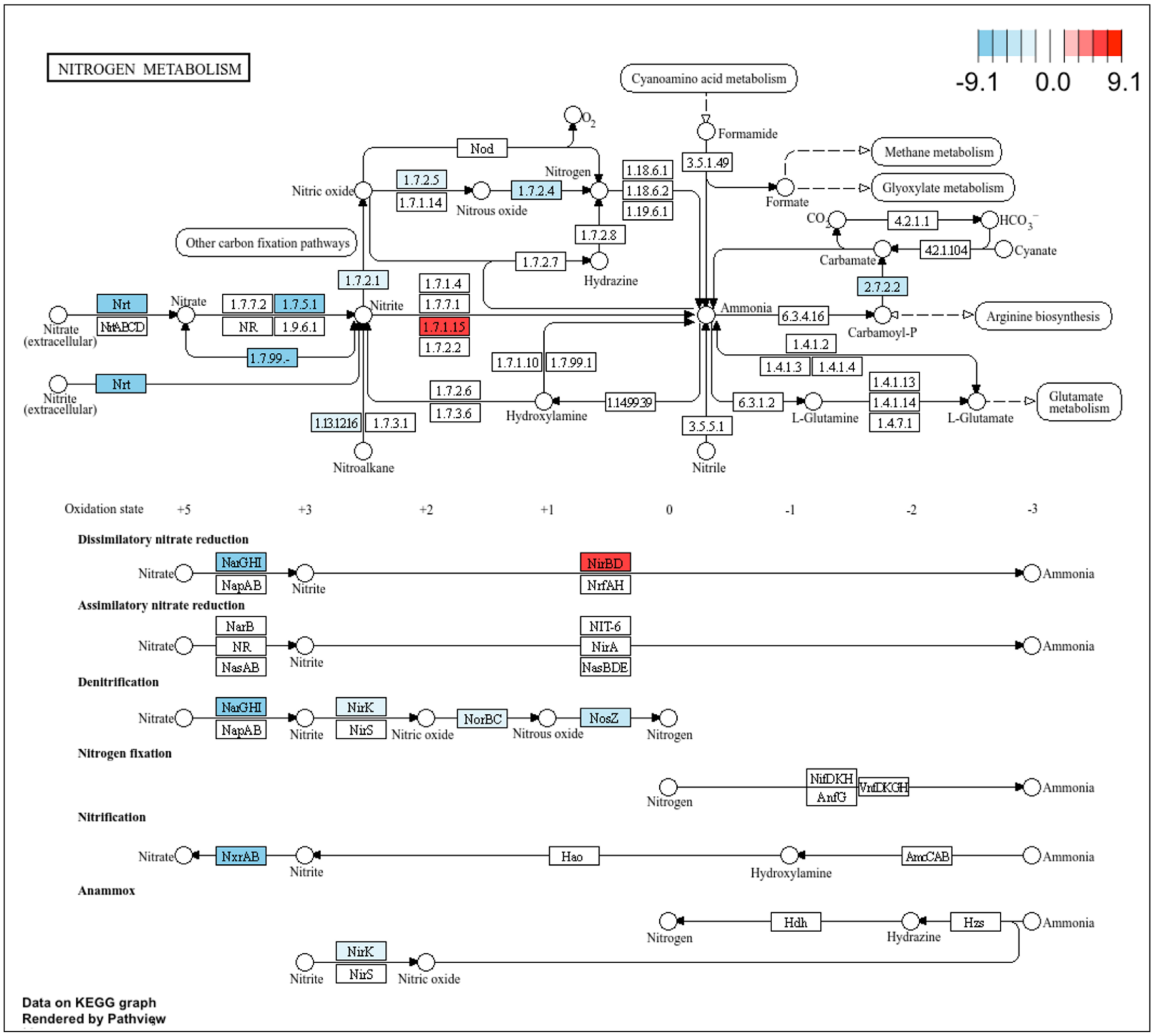

Figure S2. KEGG pathways for nitrogen metabolism. KEGG pathways for different aspects of nitrogen metabolism; see https://www.genome.jp/pathway/bte00910 for identification of locus tags. The connection between gene products (rectangles) and chemical compounds (circles) are indicated. DEGs that meet the log2-fold change of |2| are shown in color. The color-coded scale in the top-right corner represents the log2-fold changes in gene expression in stationary phase, with shades of red reflecting upregulation and shades of blue representing downregulation.

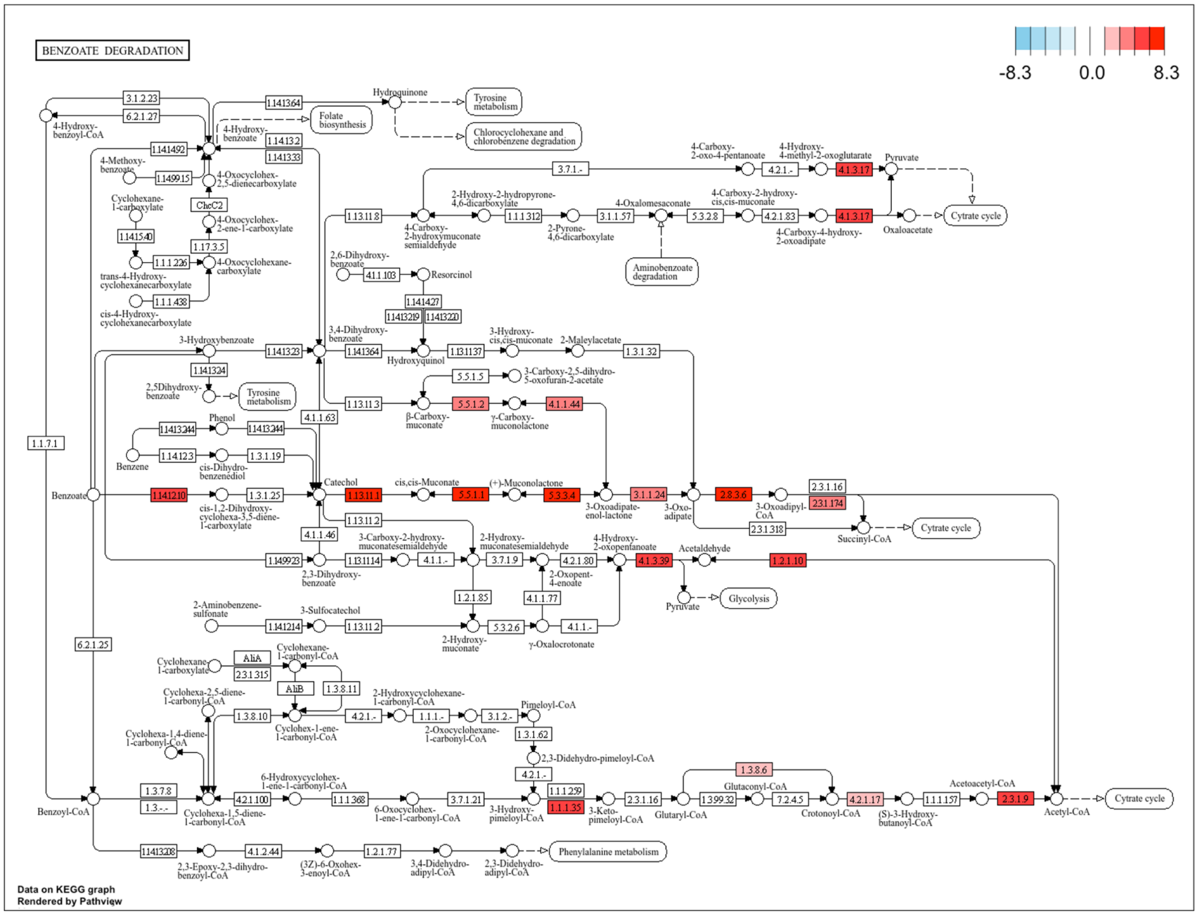

Figure S3. KEGG pathway for benzoate degradation. See https://www.genome.jp/pathway/bte00362 for identification of locus tags. The connection between gene products (rectangles) and chemical compounds (circles) are indicated. DEGs that meet the log2-fold change of |2| are shown in color. The color-coded scale in the top-right corner represents the log2-fold changes in gene expression in stationary phase, with shades of red reflecting upregulation and shades of blue representing downregulation.

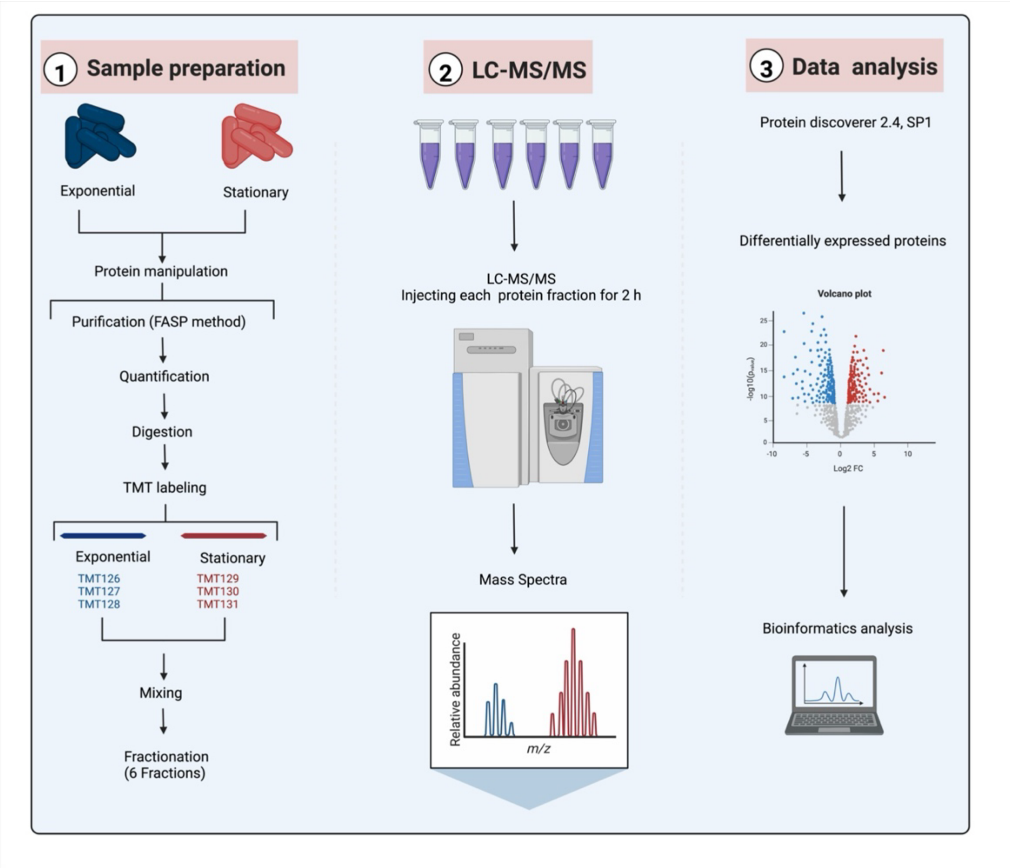

Figure S4. Mass spectrometry quantitative proteomics workflow. 1. The samples underwent protein manipulations including purification using the Filter-Aided Sample Preparation (FASP) method, protein quantification, and enzymatic digestion. The resulting peptides were labeled with Tandem Mass Tags (TMT) to enable multiplexed quantification. TMT126, TMT127, and TMT128 were used for labeling samples from the exponential phase, while TMT129, TMT130, and TMT131 were used for the stationary phase. The labeled samples were then mixed and separated into six fractions. 2. Each fraction was subjected to liquid chromatography-tandem mass spectrometry (LC-MS/MS). Peptides were separated based on their mass-to-charge ratio (*m/z*) and quantified. 3. The mass spectrometry data were processed using the Protein Discoverer 2.5 software to determine differential accumulation of proteins followed by bioinformatics analysis. Created with BioRender.com.

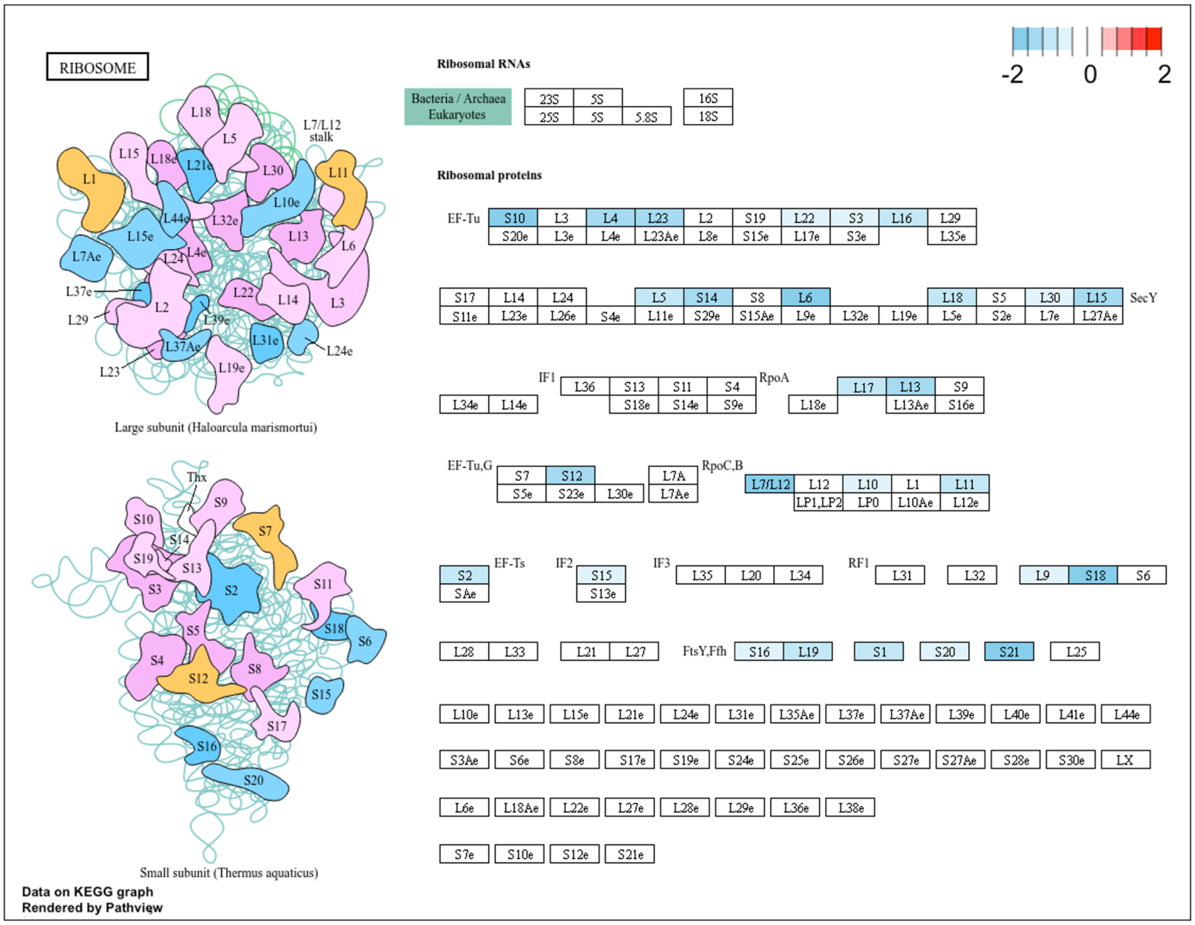

Figure S5. KEGG pathway for ribosomal proteins. A schematic representation of the ribosome (left), identifying ribosomal proteins. Ribosomal proteins and translation factors are depicted at the right; see https://www.genome.jp/pathway/bte03010 for identification of locus tags. DEPs that meet the log2-fold change of |0.5| are shown in color. The color-coded scale in the top-right corner represents the log2-fold changes in gene expression in stationary phase, with shades of red reflecting upregulation and shades of blue representing downregulation.

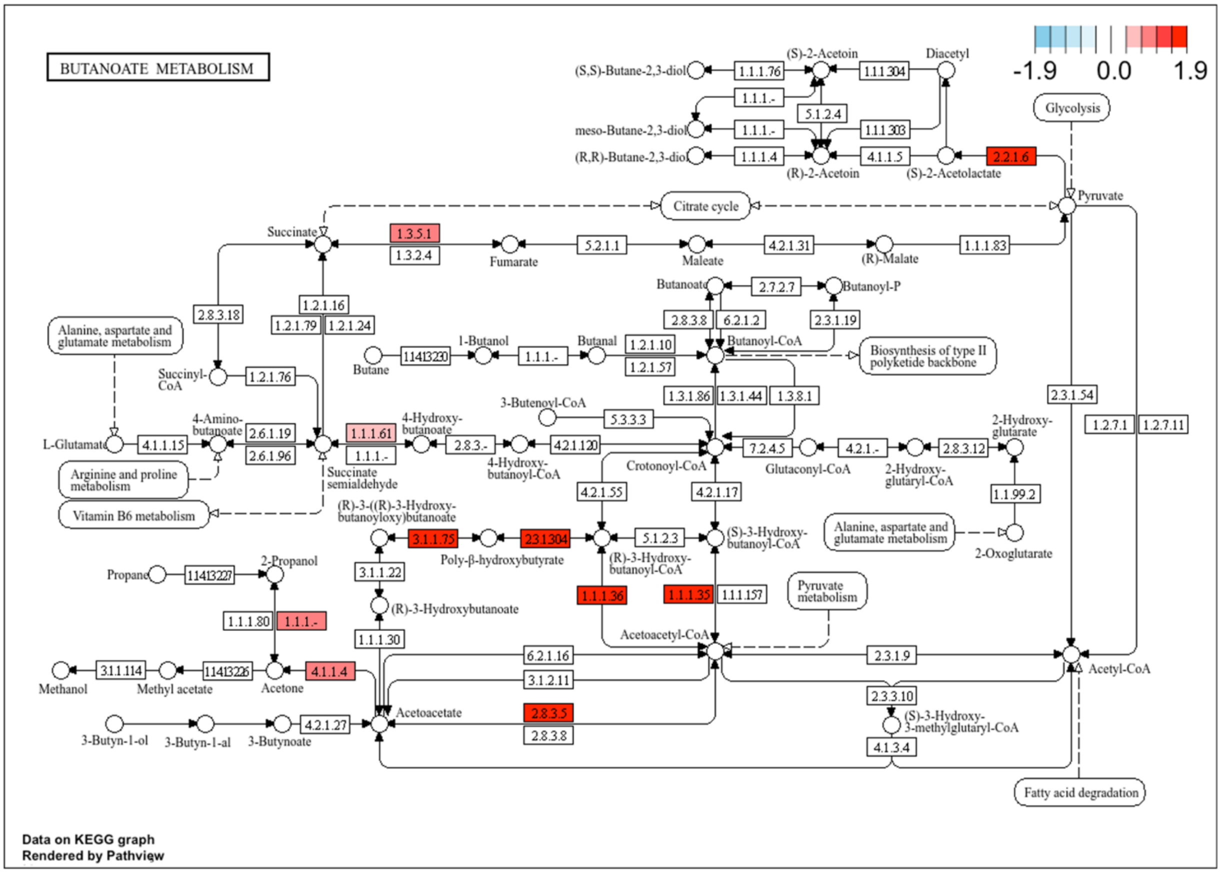

Figure S6. KEGG pathway for butanoate metabolism. See https://www.genome.jp/pathway/bte00650 for identification of locus tags. The connection between gene products (rectangles) and chemical compounds (circles) are indicated. DEPs that meet the log2-fold change of |0.5| are shown in color. The color-coded scale in the top-right corner represents the log2-fold changes in gene expression in stationary phase, with shades of red reflecting upregulation and shades of blue representing downregulation.

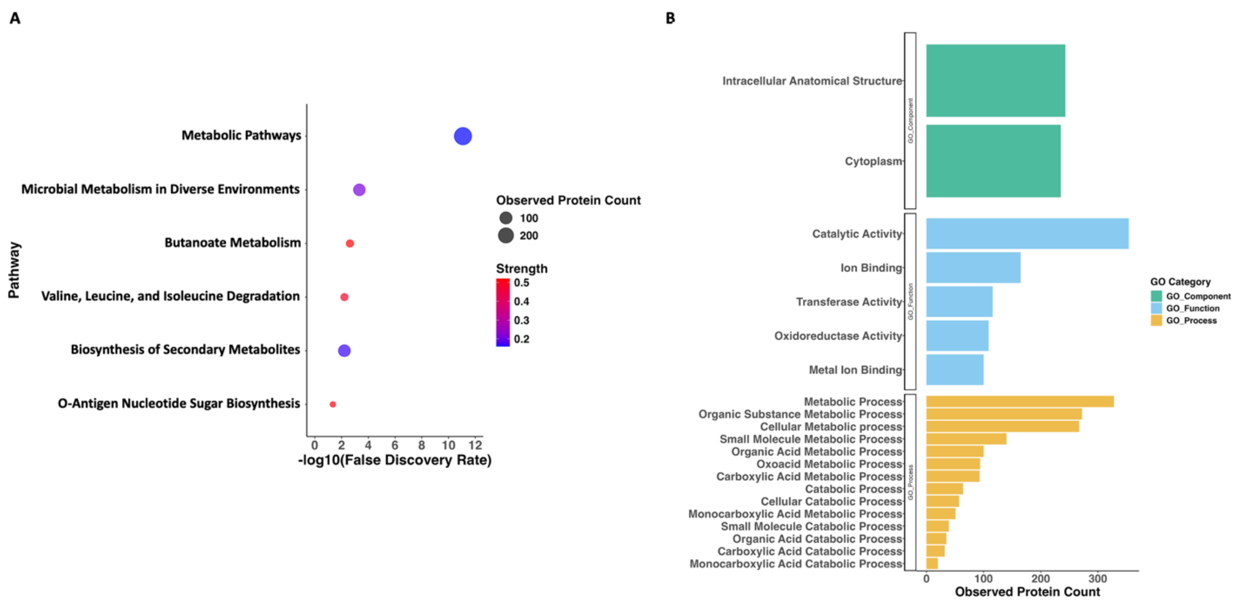

Figure S7. Protein-protein interaction (PPI) network functional clustering and GO enrichment. A. Bubble plot identifying the KEGG pathways associated with the PPI network during the stationary phase. The x-axis lists the pathway descriptions, while the y-axis represents the -log10(p-value) of the enrichment. Each bubble represents a pathway, with the size denoting the proportion of proteins in the pathway that are differentially expressed. The color gradient of the bubbles indicates the enrichment strength, with red shades representing stronger enrichment. B. GO enrichment analysis of upregulated proteins within the PPI network. The x-axis indicates the observed gene count associated with each GO term, and the y-axis lists the descriptions of the enriched terms. The plot distinguishes between different GO categories using color: Biological process (yellow), molecular function (blue), and cellular component (green).

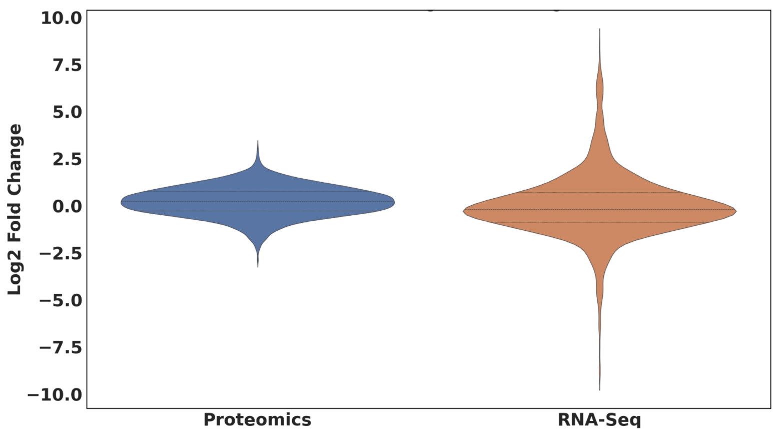

Figure S8. Violin plots for DEGs and DEPs. Distribution of log2-fold changes for proteomics (blue) and RNA-Seq (orange) datasets. Each violin plot provides a kernel density estimation of the data. For the proteomics dataset, the median log2-fold change is 0.21, with an interquartile range (IQR) of 1.04. The 95% confidence interval for the mean log2-fold change ranges from 0.19 to 0.25. The RNA-Seq dataset has a median log2-fold change of -0.2, with a broader interquartile range (IQR) of 1.6. The 95% confidence interval for the mean log2-fold change ranges from -0.09 to 0.04.

Supplemental Tables:

Table S6. Inverse expression pattern of genes and proteins.

Gene ID	Gene Log2 Fold Change	Corresponding Protein ID	Protein Log2 Fold Change	Name of protein
BTH_I2387	-4.6	Q2SVZ1	0.81	Oxidoreductase, short-chain dehydrogenase/reductase family
BTH_II1074	-2.3	Q2T6C8	0.50	HTH-type transcriptional regulator BetI
BTH_II0445	-2.2	Q2T854	0.95	ABC transporter, ATP-binding protein
BTH_I3168	-6.5	Q2STT7	0.88	Flagellar biosynthesis protein FlhF

Table S6. Inverse regulation in *B. thailandensis* stationary phase of DEGs and DEPs that meet both the log2-fold change of |2| for DEGs and |0.5| for DEPs.

Table S7. Overlap between DEPs and *B. pseudomallei* RpoS regulon.

Functional Annotation	*ΔrpoS B. pseudomallei*	Regulation in *B. thailandensis*	Identity (%)
Polyphosphate kinase 2 family (Ppk2)	Down	Up	96
Carboxymuconolactone decarboxylase (PcaC)	Down	Up	95
Nonribosomally encoded peptide/polyketide synthase (CmaB)	Down	Up	97
HSP20/alpha crystallin family protein	Down	Up	93
Universal stress protein family	Up	Up	96
Universal stress protein family	Down	Up	98
Universal stress protein UspA	Down	Up	91
Phasin (PhaP)	Down	Up	100
Alkyl hydroperoxide reductase D (AhpD)	Down	Up	94
Antioxidant, AhpC/Tsa family	Down	Up	98
Osmotically inducible Y domain protein (OsmY)	Down	Up	92
Osmotically inducible Y domain protein (OsmY)	Down	Up	95
NADPH-dependent FMN reductase	Down	Up	92
Inclusion body family protein	Down	Up	33
Ribosomal natural product, two-chain TOMM family	Down	Up	76
DUF1842 domain-containing protein	Down	Up	85
Hypothetical protein BPSS0213	Down	Up	84
Polyketide synthase, putative	Down	Up	75
4-hydroxy-3-methylbut-2-enyl diphosphate reductase (IspH)	Up	Down	99
Chaperonin GroES	Down	Down	99
Alkyl hydroperoxide reductase C	Down	Down	31

Table S7. Proteins for which the corresponding genes were found to be regulated in a *B. pseudomallei ΔrpoS* strain relative to wild-type. Regulation in *B. thailandensis* refers to stationary phase relative to exponential growth; only proteins that meet the log2-fold change of |0.5| are included.

Table S8. Overlap between DEPs and *E. coli* RpoS regulon.

ΔrpoS E. coli	**Protein annotation in *E. coli***	**Regulation in *B. thailandensis***	**Protein annotation in *B. thailandensis***	**Identity (%)**
Down	TufA - Elongation factor Tu	Up	Elongation factor Tu (EF-Tu)	80
Up	AckA - Acetate kinase	Up	Acetate kinase	36
Up	GroES - 10 kDa chaperonin	Down	Co-chaperonin GroES, 10 kDa chaperonin	53
Up	FabB – 3-oxoacyl-[acyl-carrier-protein] synthase 1	Down	3-oxoacyl-[acyl-carrier-protein] synthase 2	38

Table S8. Proteins for which the corresponding genes were found to be regulated in an *E. coli ΔrpoS* strain relative to wild-type. Regulation in *B. thailandensis* refers to stationary phase relative to exponential growth; only proteins that meet the log2-fold change of |0.5| are included.

Table S9. RT-qPCR primer sequences.

**Name**	**Sequence (5' to 3')**
RpoS_FW_BTH_I2226	ACGACATCGCGTATCTGACC
RpoS_RV_BTH_I2226	GGGTAGAAGATCGAGCAGGC
Glutamate synthase_FW_BTH_I3014	GCAAGAAGAGCCACGAAATC
Glutamate synthase_RV_BTH_I3014	CCATCTCCTCGCGATAGAAC

Detailed procedures for TMT labeling and protein fractionation

Samples were processed using the filter-aided sample preparation (FASP) method,[1] with modifications. Forty mL of the 100 mL bacterial culture were centrifuged at 17,800 × g for 5 min, followed by two washes with phosphate buffered saline (PBS). The bacterial pellets were stored at -80°C. Cell pellets were thawed on ice and protein extraction was carried out by addition of 1 mL of a lysis buffer (2% SDS in 100 mM tetraethylammonium bicarbonate (TEAB), pH 8.5). The cell pellets were sonicated twice using a Branson SFX250 Sonifier at 35% output at 10 secs pulse with 1-minute gap in between. Homogenized samples were centrifuged at 8,600 × g for 5 min at 4ºC. Protein concentration of the supernatant was determined using the Pierce™ bicinchoninic acid (BCA) Protein Assay Kit (Thermo Fisher Scientific).

Proteins (100 µg total in 100 µL) were first reduced with 50 mM DTT at 95ºC for 5 min. Samples were loaded onto 10 kDa ultrafiltration units (Sigma Aldrich) and centrifuged at 10,000 × g for 15 min. After that, 200 µl of 8 M urea was added to the ultrafiltration units, followed by centrifugation for 10,000 x g for 10 min at 4ºC. Peptides were alkylated by addition of 100 µL 50 mM iodoacetamide and incubation for 20 min at room temperature in the dark. Samples were then centrifuged at 10,000 × g for 15 min. Subsequent overnight digestion was performed using 0.5 µg μL^{-1} LysC/Trypsin (Thermo Fisher Scientific) at a 1:100 enzyme to protein ratio in digestion buffer (0.1 M TEAB, pH 8.0) at 37°C. Peptides were collected by centrifugation at 10,000 × g for 5 min followed by addition of 50 µL of digestion buffer and centrifugation at 10,000 × g. The 100 µg starting protein quantity was chosen based on the published protocol[1] and past experience. Generally, a loss of up to 50% of the sample is associated with this protocol in a tradeoff for a high peptide sample purity. Accordingly, the generated tryptic digests provided ~50 µg of peptide material for subsequent TMT tagging.

TMTsixplex™ labeling was conducted following the manufacturer's protocol (Thermo Fisher Scientific). Peptides (three samples representing exponential phase and three from stationary phase) were lyophilized and resuspended in 26 µL TEAB buffer (pH 8.5). The six isobaric TMT reagents were equilibrated to room temperature and reconstituted in 41 µL anhydrous acetonitrile (ACN), vortexed intermittently for 5 min, and centrifuged briefly. For each of the six peptide samples, 10 µL of a distinct TMT reagent was added to 35 µg peptide sample, and the reaction volume was brought to 36 µL using the TEAB buffer before incubation at room temperature for 1 h. These volumes were chosen to replicate the same concentration used in the manufacturer's protocol, but with a lower volume.[2] The reactions were quenched with 2 µL 5% hydroxylamine, and equal amounts of labeled peptides from all six conditions (5 µg each) were pooled and stored at -20ºC.

Samples were fractionated using strong cation exchange stage tips (AttractSPE Disk, Affinisep, Le Houlme, Normandy, France). The tips were conditioned with 200 µL of ACN twice, followed by equilibration with 200 µL of 0.1% formic acid in water. These tips require 30 µg of total peptide material. Therefore, we used 5 µg of each TMT reaction for pooling to generate the required 30 µg total for fractionation. Peptides (30 µg total) were loaded onto the tip and washed twice with 200 µL of 0.2% formic acid in water. Fractionation was performed using a stepwise elution with ammonium acetate (NH_4OAc, pH 3.0) in 0.1% formic acid (FA), with the following conditions (200 µL of each solution): Fraction 1: 50 mM NH_4OAc, 20% ACN, 0.5% FA; Fraction 2: 75 mM NH_4OAc, 20% ACN, 0.5% FA; Fraction 3: 125 mM NH_4OAc, 20% ACN, 0.5% FA; Fraction 4: 200 mM NH_4OAc, 20% ACN, 0.5% FA; Fraction 5: 300 mM NH_4OAc, 20% ACN, 0.5% FA; Fraction 6: 5% ammonium hydroxide, 80% ACN. Each of the six fractions were dried under vacuum and resuspended in 10 µL 0.1% formic acid prior to LC-MS/MS analysis.

For mass spectrometric analysis, one-half of the reconstituted peptide solution (5 µL) was injected into the Orbitrap Q-Exactive mass spectrometer. Following data acquisition, raw spectra from all six fractions of each sample were processed using Proteome Discoverer software,[3] where the individual fractionated datasets were merged to generate a single comprehensive file representing the entire experiment, ensuring a unified representation of the proteomic profile.

Detailed procedures for statistical analyses

Dataset overview

Transcriptomic (RNA-Seq) and proteomic datasets were obtained from *B. thailandensis* E264 cultures to compare the exponential growth and stationary phases. To ensure a direct comparison, both RNA and protein were extracted from the same biological samples, allowing us to integrate mRNA and protein expression levels without biological variability. Each condition had three biological replicates.

Data preprocessing and normalization

Preprocessing and filtering was performed to retain only the entries detected at both the transcript and protein levels. For expression changes, we used log2 transformation, which symmetrizes the distribution. Because RNA-Seq captures nearly the full transcriptome, we set a stringent cutoff for significant differential expression: |log2 fold change| ≥ 2. Proteomic data do not encompass the entire proteome due to inherent limitations in mass spectrometry coverage and detection, necessitating a more lenient threshold of $|\log_2$ fold change$| \geq 0.5$.

To make transcript and protein changes directly comparable, we normalized both datasets using z-score scaling (standard score normalization).[4] The z-score transformation for each data point x_i was calculated using the formula:

$$Z_i = \frac{x_i - \mu}{\sigma}$$

where:

x_i = log2 fold-change value for a given transcript or protein.
μ = mean log2 fold change of the dataset.
σ = standard deviation of log2 fold changes.

Statistical analysis

To quantify the relationship between transcript and protein expression changes, we used Pearson correlation analysis on the integrated dataset.[5] The Pearson correlation coefficient (r) was calculated using the formula:

$$r = \frac{n \sum xy - (\sum x)(\sum y)}{\sqrt{(n \sum x^2 - (\sum x)^2) . (n \sum y^2 - (\sum y)^2)}}$$

where:

n = total number of paired transcript-protein measurements in the merged dataset.
x = log2 fold-change values from RNA-Seq.
y = log2 fold-change values from Proteomics.

The correlation coefficient (r) was calculated using the SciPy library pearsonr function,[6] which also returns a p-value to determine statistical significance. The p-value tests the null hypothesis that $r=0$ (no correlation). We set a significance threshold of $\alpha = 0.05$.

Reproducibility and GitHub Repository

To ensure full reproducibility, the complete data integration and statistical analysis script is available on GitHub:
GitHub Repository: https://github.com/Ahmed-Tohamy/rna_proteomics_integration

References

(1) Wiśniewski, J. Filter-aided sample preparation: the versatile and efficient method for proteomic analysis. In *Methods in enzymology*, Vol. 585; Elsevier, 2017; pp 15-27.
(2) Zecha, J.; Satpathy, S.; Kanashova, T.; Avanessian, S. C.; Kane, M. H.; Clauser, K. R.; Mertins, P.; Carr, S. A.; Kuster, B. TMT Labeling for the Masses: A Robust and Cost-efficient, In-solution Labeling Approach. *Mol Cell Proteomics* **2019**, *18* (7), 1468-1478. DOI: 10.1074/mcp.TIR119.001385 From NLM Medline.
(3) Osburn, B. C. Proteome discoverer—a community enhanced data processing suite for protein informatics. *Proteomes* **2021**, *9*, 15.
(4) Larsen, R. J.; Marx, M. L. *An introduction to mathematical statistics*; Prentice Hall, 2005.
(5) Pearson, K. Note on regression and inheritance in the case of two parents. *Proc Royal Soc London* **1895**, *58*, 240-242.
(6) Virtanen, P.; Gommers, R.; Oliphant, T. E.; Haberland, M.; Reddy, T.; Cournapeau, D.; Burovski, E.; Peterson, P.; Weckesser, W.; Bright, J.; et al. SciPy 1.0: fundamental algorithms for scientific computing in Python. *Nat Methods* **2020**, *17* (3), 261-272. DOI: 10.1038/s41592-019-0686-2 From NLM Medline.
